# Experimental Models and Nanotechnology-Based Platforms in Oral Squamous Cell Carcinoma: From Tumor Biology to Translational Applications

**DOI:** 10.3390/pharmaceutics18080995

**Published:** 2026-08-12

**Authors:** Patricia Rodríguez Carballido, João P. N. Silva, Andrea Cunha, Patrícia M. A. Silva

**Affiliations:** 1 UNIPRO—Oral Pathology and Rehabilitation Research Unit, University Institute of Health Sciences (IUCS)-CESPU, 4585-116 Gandra, Portugal; patitavrc7@gmail.com (P.R.C.); joaosilva_06@hotmail.com (J.P.N.S.); 2 Associate Laboratory i4HB, Institute for Health and Bioeconomy, University Institute of Health Sciences (IUCS)-CESPU, 4585-116 Gandra, Portugal; 3 UCIBIO—Applied Molecular Biosciences Unit, Translational Toxicology Research Laboratory, University Institute of Health Sciences (1H-TOXRUN, IUCS)-CESPU, 4585-116 Gandra, Portugal

**Keywords:** oral squamous cell carcinoma, experimental models, metabolic reprogramming, tumor microenvironment, nanotechnology, translational research

## Abstract

Oral cancer, predominantly represented by oral squamous cell carcinoma (OSCC), remains a major global health burden due to its aggressive clinical behavior, high recurrence rates, and limited improvement in survival over recent decades. Despite advances in treatment modalities, patient outcomes remain poor largely due to late diagnosis, therapeutic resistance, and profound tumor heterogeneity. In particular, metabolic reprogramming has emerged as a central hallmark of oral carcinogenesis, enabling tumor cells to adapt to hypoxic and nutrient-deprived microenvironments while promoting proliferation, invasion, and treatment resistance. Traditional experimental models, including two-dimensional cell cultures and in vivo animal models, have provided important mechanistic insights. However, they fail to fully recapitulate the metabolic, structural, and cellular complexity of human tumors. Consequently, there is growing interest in more physiologically relevant platforms, such as three-dimensional spheroids, organoids, and patient-derived models, which better preserve tumor architecture and microenvironmental interactions. In parallel, multi-omics approaches are increasingly being integrated to dissect the molecular and metabolic complexity of oral cancer at unprecedented resolutions, while nanotechnology-based systems are emerging as promising tools for targeted drug delivery and improved therapeutic precision. This review discusses experimental models in oral cancer research, focusing on their ability to capture metabolic alterations and tumor heterogeneity. We further highlight their translational potential together with multi-omics integration and nanotechnology-based strategies for improving biomarker discovery, therapeutic stratification, and the development of more effective treatment approaches.

## 1. Introduction

According to GLOBOCAN 2022, oral cancer is responsible for more than 389,846 new cases and 188,438 deaths each year worldwide, ranking as the 16th most frequently diagnosed cancer globally [1]. The data reveal a significant male predominance in both the incidence and mortality of cancers of the lip and oral cavity [1]. In 2022, there were an estimated 268,759 new cases in men compared to 120,726 in women [1]. Consistent with many solid tumors, the risk of developing oral cancer tends to increase with age, as a result of cumulative exposure to risk factors (Section 2.1) and the demographic transition of populations [1]. Within this disease group, oral squamous cell carcinoma (OSCC) represents the predominant histological subtype and remains a major clinical challenge due to its aggressive behavior, high recurrence rates, and persistently poor survival outcomes [2].

Despite advances in surgical techniques, radiotherapy, chemotherapy, immunotherapy, and targeted therapies—including epidermal growth factor receptor (EGFR) and cyclooxygenase-2 inhibitors—long-term survival has improved only modestly over recent decades [3].

At the molecular level, oral carcinogenesis is driven by a complex interplay of genetic (e.g., *TP53*, *CDKN2A*, and *PIK3CA* dysregulation), epigenetic modifications (including aberrant DNA methylation and histone modifications), and metabolic reprogramming that collectively shape tumor initiation and progression [4]. Among these, metabolic reprogramming has emerged as a central adaptive mechanism that enables tumor cells to thrive under hypoxic and nutrient-deprived conditions while sustaining uncontrolled proliferation, facilitating local invasion, and promoting resistance to therapy [5]. In parallel, marked inter- and intra-tumoral heterogeneity, reflecting differences between tumors from different patients, and intratumoral heterogeneity, characterized by the coexistence of diverse cellular subpopulations within the same tumor, contribute to metabolic adaptation, tumor progression, and treatment failure [6]. The tumor microenvironment (TME) plays a pivotal role in orchestrating these processes, regulating immune evasion, stromal remodeling, and the emergence of therapy-resistant phenotypes [7].

Together, these interconnected biological features highlight the limitations of conventional experimental systems in faithfully reproducing the complexity and dynamic nature of human oral tumors [8]. Traditional in vitro models, such as two-dimensional (2D) cell cultures and in vivo animal models, have been instrumental in advancing our understanding of oral cancer biology because of their simplicity, reproducibility, and cost-effectiveness [8]. However, these systems incompletely reproduce the three-dimensional (3D) architecture of tumors, the cellular heterogeneity of the TME, dynamic cell–cell and cell–extracellular matrix interactions, and the metabolic gradients that critically influence tumor progression and therapeutic response [8]. Consequently, more physiologically relevant experimental platforms, including 3D spheroids, organoids, organ-on-chip (OoC) systems, and patient-derived models, have emerged as complementary approaches that better preserve tissue architecture, tumor heterogeneity, and clinically relevant biological features [8]. While these advanced models offer greater physiological relevance and translational potential, they are generally associated with increased technical complexity, higher costs, and lower experimental throughput compared with conventional systems [8]. Therefore, rather than replacing traditional models, they should be viewed as complementary platforms whose selection depends on specific biological questions and research objectives [8].

This review provides a critical overview of conventional and advanced experimental models used in oral cancer research, discussing their respective strengths and limitations, as well as their suitability for addressing distinct aspects of tumor metabolism, heterogeneity, and therapeutic response. Furthermore, it highlights how an integration of these platforms with multi-omics approaches and nanotechnology-based strategies may contribute to bridging the persistent gap between experimental research and clinical translation [9,10].

## 2. Oral Squamous Cell Carcinoma

### 2.1. Epidemiology, Clinical Features, and Therapeutic Challenges

OSCC is a malignant epithelial neoplasm arising from the stratified squamous epithelium lining the oral cavity [11]. It is characterized by uncontrolled proliferation of squamous cells, local tissue invasion, and the potential for regional and distant metastasis [4]. OSCC may affect several anatomical sites within the oral cavity, including the tongue, floor of the mouth, buccal mucosa, gingiva, palate, and lips [4]. OSCC accounts for approximately 90% of all oral malignancies and represents a major global health burden [12]. Despite advances in diagnostic techniques and therapeutic strategies, the overall 5-year survival rate for OSCC remains approximately 50–60%, largely due to late-stage diagnosis, high rates of local invasion, and frequent recurrence [13]. Risk factors for oral cancer, including OSCC, remain primarily associated with environmental and behavioral exposures [14]. Tobacco use is the most established risk factor, showing a strong dose-dependent effect and a well-documented synergistic interaction with alcohol consumption, which enhances mucosal permeability and carcinogen exposure [11]. Alcohol alone is also an independent risk factor, particularly in chronic and heavy use cases [13,15,16]. Human papillomavirus (HPV), especially HPV-16, is strongly linked to oropharyngeal cancers, although its role in oral cavity tumors is less consistent [17]. Additional risk factors include poor oral hygiene, chronic mucosal inflammation, low intake of fruits and vegetables, and exposure to occupational or environmental carcinogens [11]. Emerging evidence also highlights the potential role of oral microbiome dysbiosis in promoting a pro-inflammatory and pro-tumorigenic microenvironment [11,18]. Clinically, OSCC is characterized by aggressive local invasion and a strong propensity for regional lymph node metastasis, which significantly impacts prognosis and survival outcomes [13]. Treatment follows a stage-dependent multimodal approach. Early-stage disease is primarily managed with surgical resection, often followed by adjuvant radiotherapy in the presence of adverse pathological features [19]. Locally advanced disease typically requires combined chemoradiotherapy, with cisplatin (CDDP)-based regimens representing the standard of care [20]. In recurrent or metastatic OSCC, immune checkpoint inhibitors (ICIs) targeting the programmed cell death protein 1 (PD-1)/programmed death-ligand 1 (PD-L1) axis, such as pembrolizumab and nivolumab, have become established therapeutic options either as a monotherapy or in combination with chemotherapy, depending on PD-L1 expression and clinical context [21,22]. EGFR-targeted therapies, particularly cetuximab, may be used in selected cases, either alone or combined with platinum-based chemotherapy, although their clinical benefit remains more limited compared with immunotherapy-based regimens [23]. Overall, treatment strategies are increasingly individualized based on tumor stage, performance status, and molecular features, as reflected in contemporary clinical guidelines [19]. However, despite therapeutic advances, resistance to treatment and tumor recurrence remain frequent in OSCC, underscoring persistent clinical challenges and the need for improved predictive models and personalized therapeutic strategies [24].

### 2.2. Metabolic Reprogramming, Tumor Heterogeneity, and Therapeutic Resistance in Oral Squamous Cell Carcinoma

**Figure 1:**
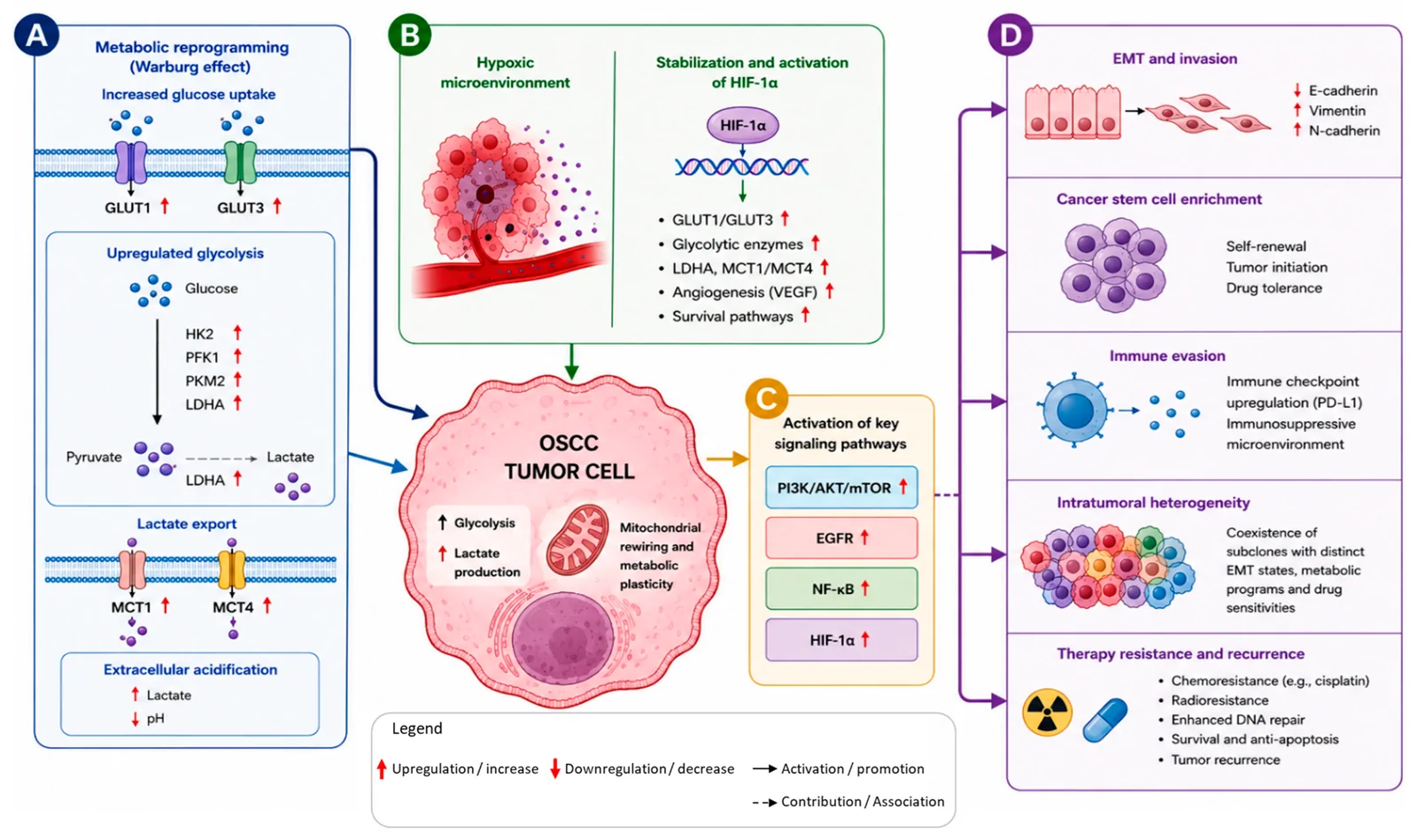
Interplay between metabolic reprogramming, tumor heterogeneity, and therapeutic resistance in OSCC. Metabolic rewiring in OSCC is characterized by: (**A**) enhanced glycolysis, increased glucose uptake, lactate accumulation; and (**B**) activation of hypoxia-associated pathways; (**C**) the key signaling pathways involved include PI3K/AKT/mTOR, EGFR, NF-κB, and HIF-1α; and (**D**) these alterations promote EMT, cancer stem cell maintenance, immune evasion, and intratumoral heterogeneity, collectively contributing to tumor progression and resistance to chemotherapy and radiotherapy. This figure was created using a generative AI tool based on an author-provided conceptual description and subsequently reviewed and edited by the authors. EGFR, epidermal growth factor receptor; EMT, epithelial–mesenchymal transition; GLUT, glucose transporter; HIF-1α, hypoxia-inducible factor-1α; HK2, hexokinase 2; LDHA, lactate dehydrogenase A; MCT, monocarboxylate transporter; PD-L1, programmed death-ligand 1; PFK1, phosphofructokinase 1; PKM2, pyruvate kinase M2; VEGF, vascular endothelial growth factor.

Metabolic reprogramming is recognized as a central hallmark of cancer biology and reflects the ability of the tumor to adapt to a hypoxic and nutrient-deprived microenvironment [25]. Enhanced aerobic glycolysis, known as the Warburg effect, increased lactate production, alterations in lipid metabolism, and mitochondrial rewiring have been widely reported in OSCC and are strongly associated with tumor progression and therapy resistance [26]. Increased expression of glucose transporters, such as glucose transporter (GLUT) 1 and GLUT3, together with the upregulation of glycolytic enzymes, including pyruvate kinase M2, lactate dehydrogenase A, and members of the phosphofructokinase family, further supports the metabolic dependency of OSCC cells on glycolysis under both normoxic and hypoxic conditions [27,28,29] (Figure 1). In parallel, the overexpression of hypoxia-responsive regulators, such as hypoxia-inducible factor-1α (HIF-1α) and monocarboxylate transporters (MCT1/MCT4), contributes to lactate accumulation, extracellular acidification, and adaptation to TME [30,31,32,33,34,35]. These metabolic adaptations not only sustain rapid proliferation but also promote epithelial–mesenchymal transition (EMT), immune evasion, and cancer stem cell-like phenotypes [30,31,32,33,34,35]. EMT plays a central role in OSCC progression characterized by the loss of epithelial markers, such as E-cadherin, and the gain of mesenchymal markers, including vimentin and N-cadherin, facilitating invasion and metastasis [36,37,38]. Furthermore, EMT has been closely linked to chemoresistance, particularly to CDDP, through the activation of survival pathways, such as PI3K/AKT/mTOR, and the upregulation of drug efflux transporters [39,40]. Importantly, metabolic plasticity enables tumor cells to switch between glycolytic and oxidative phosphorylation states depending on environmental conditions, reinforcing therapeutic resistance [41]. In parallel, OSCC exhibits pronounced molecular heterogeneity at genomic, transcriptomic, epigenetic, and metabolic levels. Large-scale sequencing studies have identified recurrent alterations in tumor protein p53 (*TP53*), cyclin-dependent kinase inhibitor 2A (*CDKN2A*), FAT Atypical Cadherin 1 (*FAT1*), Notch homolog 1, translocation-associated (*NOTCH1*), and PI3K/AKT/mTOR signaling components, although substantial variability exists among patients and even among different regions of the same tumor [42,43]. More recently, single-cell RNA sequencing (RNA-seq) and spatial transcriptomic approaches demonstrated the coexistence of multiple malignant cellular subpopulations with distinct proliferative capacities, EMT states, metabolic programs, and drug sensitivities within the same TME [6,44]. Therapy resistance in OSCC arises from multiple interconnected mechanisms, including the activation of survival pathways, metabolic adaptation, enhanced DNA repair capacity, hypoxia-associated signaling, and dynamic interactions with the TME [42,45,46]. Activation of the PI3K/AKT/mTOR, EGFR, NF-κB, and HIF-1α signaling pathways further promotes apoptosis evasion and resistance to chemotherapy and radiotherapy [42,45,46]. Moreover, cancer stem cell (CSC) enrichment, immune checkpoint activation, stromal remodeling, and hypoxic niche formation contribute to reduced therapeutic sensitivity and tumor recurrence [47].

## 3. In Vitro Models to Study Oral Squamous Cell Carcinoma

### 3.1. Conventional Two-Dimensional Cultures

Two-dimensional monolayer cultures remain widely used in OSCC research due to their simplicity, reproducibility, and compatibility with molecular, biochemical, and functional assays [48]. Established OSCC cell lines derived from primary tumors or metastatic lesions, including SCC-9, SCC-15, SCC-25, CAL-27, HSC-2, HSC-3, HSC-4, and SAS, provide standardized platforms for investigating tumor progression and treatment responses [49,50,51,52,53,54,55]. Among these models, HSC-3 and SAS cell lines are frequently used to investigate invasive behavior and metastasis-associated mechanisms due to their highly aggressive phenotype [56,57,58]. These cell lines typically exhibit increased expression of EMT-associated markers, including vimentin and matrix metalloproteinase (MMP)-2/9, together with reduced E-cadherin expression, reflecting enhanced migratory and invasive capacity [59]. Similarly, SCC-9 and CAL-27 cell lines are widely used to investigate proliferation, epithelial signaling pathways, therapeutic response, and metabolic adaptation, although they are also suitable for studies of cell migration and invasion [52,54]. CAL-27 cells contributed to the characterization of metabolic alterations in OSCC and demonstrated increased glycolytic activity and mitochondrial dysfunction compared with normal oral keratinocytes [60]. In addition, transcriptomic and metabolic analyses identified an increased expression of glucose metabolism-related markers, including GLUT1, suggesting enhanced glucose uptake and metabolic adaptation to the increased energetic requirements of tumor cells [58,61,62]. Beyond metabolic characterization, SCC-25 and CAL-27 cells have been widely used in preclinical studies evaluating the therapeutic strategies targeting oncogenic and metabolic pathways. These models have been employed to investigate responses to EGFR inhibitors, PI3K/AKT/mTOR pathway inhibitors, glycolysis-targeting compounds, such as 2-deoxyglucose (2-DG), and platinum-based chemotherapy, including CDDP [63,64]. Such studies have provided insights into mechanisms underlying treatment resistance, including the activation of survival signaling pathways and hypoxia-induced adaptation, and the modulation of reactive oxygen species (ROS) [63,64]. Extracellular matrix (ECM)-1, a well-characterized OSCC cell line derived from buccal mucosa, has also been extensively used to investigate tumor progression, signaling pathways, and chemoresistance [65,66,67]. Studies using this model demonstrated the involvement of p53/p21 and EGFR/PI3K/AKT signaling in malignant progression, as well as the contribution of inflammatory mediators, including IL-1β, to therapy resistance and tumor–microenvironment interactions [65,66,67]. Two-dimensional cultures are readily compatible with advanced experimental approaches, including CRISPR/Cas9 genome editing, transcriptomics, proteomics, metabolomics, and Seahorse-based metabolic flux analysis, enabling a detailed investigation of tumor biology, metabolic regulation, and therapeutic response [68]. Despite these advantages, the ability of 2D models to reproduce the complexity of OSCC metabolism remains limited. Their uniform exposure to oxygen and nutrients prevents the establishment of physiologically relevant gradients, while the absence of ECM organization, stromal components, and immune interactions restricts the modeling of tumor heterogeneity and microenvironment-driven metabolic adaptation [69].

### 3.2. Three-Dimensional Spheroid Cultures

3D culture systems represent a significant advancement over conventional 2D models by more closely recapitulating the structural, functional, and metabolic organization of solid tumors [70]. In OSCC, multicellular spheroids preserve physiologically relevant cell–cell and cell–ECM interactions while establishing oxygen, nutrient, and metabolite gradients that generate proliferative peripheral layers surrounding hypoxic or necrotic cores [71,72]. This architecture has been experimentally demonstrated in CAL-27 and HSC-3 spheroids through pimonidazole staining to identify hypoxic regions, together with increased HIF-1α expression within the spheroid core [73,74]. The presence of these gradients enables the study of intratumoral metabolic heterogeneity and hypoxia-driven adaptive responses that are not reproduced in conventional monolayer cultures [75,76]. In particular, HIF-1α activation promotes transcriptional programs associated with glycolytic metabolism, angiogenesis, and cellular adaptation to low oxygen availability, making spheroids a valuable platform for investigating the metabolic plasticity of OSCC cells [76,77,78]. Importantly, recent studies have demonstrated the value of integrating 3D spheroid cultures with functional metabolic analyses. Using four established oral carcinoma cell lines representing different histopathological grades (three OSCC and one oral adenosquamous carcinoma), Miyamoto et al. demonstrated that increased ATP-linked respiration and reduced proton leak, determined by Seahorse extracellular flux analysis in 2D cultures, were associated with higher pathological malignancy [75]. Moreover, the corresponding 3D spheroids displayed distinct morphologies that paralleled cisplatin sensitivity, suggesting that mitochondrial bioenergetics and spheroid architecture may serve as complementary indicators of tumor aggressiveness and therapeutic response. Similarly, Miao et al. combined multicellular tumor spheroids enriched for CSC-like populations derived from CAL27 and HSC3 OSCC cell lines with metabolomic, transcriptomic, and single-cell RNA-seq analyses [79]. These integrated approaches revealed metabolic differences between CSC-like and differentiated cancer cells, involving pathways related to glycolysis, the tricarboxylic acid cycle, fatty acid oxidation, glutathione metabolism, and glycerophospholipid metabolism. These findings support the use of spheroid models to investigate metabolic heterogeneity associated with CSC-like populations in OSCC [79]. OSCC spheroids are predominantly generated using ultra-low attachment (ULA) or hanging-drop methods, while matrix-supported systems based on collagen, Matrigel, or hydrogels further enhance physiological relevance by reproducing ECM-derived biochemical and biomechanical cues [62,71,73]. More advanced co-culture and microfluidic platforms integrate fibroblasts, endothelial, and immune cells, enabling the investigation of stromal interactions involved in invasion, EMT, angiogenesis, and therapy resistance [59,62]. Although current 3D models remain limited by variability in spheroid size, incomplete vascularization, and the lack of fully functional immune components, they constitute an essential intermediate platform between conventional monolayer cultures and in vivo models [80].

### 3.3. Patient-Derived Organoids

Patient-derived organoids (PDOs) have emerged as an advanced preclinical platform for modeling OSCC while preserving patient-specific histopathological features, genomic alterations, and intratumoral heterogeneity [81,82,83,84]. Compared with conventional cell lines, PDOs more faithfully reproduce the molecular landscape of head and neck squamous cell carcinoma (HNSCC), including frequent alterations in *TP53*, *FAT1*, *CDKN2A*, and PI3K/AKT signalling pathways [42,55]. Recent studies have demonstrated the successful establishment of OSCC PDO biobanks from surgical specimens, maintaining tissue architecture, differentiation status, and genomic profiles across serial passages, while reproducing the heterogeneous therapeutic responses observed in patients [67,84]. Beyond preserving tumor heterogeneity, PDOs have become valuable tools for functional studies of tumor metabolism and therapeutic response. Zhao et al. demonstrated that lactate promoted CSC-like properties in OSCC organoids by increasing CD133 expression through MCT1-dependent lactate transport, whereas pharmacological inhibition of MCT1 reversed these effects, identifying lactate metabolism as a potential therapeutic target [85]. Similarly, Wang et al. showed that the disruption of *lysine methyltransferase 2D* (*KMT2D*)-*myocyte enhancer factor 2A* (*MEF2A*) signalling reduced stem-like properties and organoid-forming capacity, supporting the role of epigenetic regulation in maintaining aggressive tumor phenotypes [86]. PDOs have also facilitated the investigation of tumor–stroma interactions. Using a 3D co-culture model, Zhao et al. demonstrated that cancer-associated fibroblasts (CAFs) enhanced organoid formation and stemness through stromal-derived signaling, highlighting the importance of the TME in OSCC progression [87]. From a translational perspective, PDOs have shown considerable promise for precision oncology. Millen et al. demonstrated that patient-derived HNSCC organoids reproduced individual responses to radiotherapy, CDDP, cetuximab, and other targeted therapies, supporting their application for treatment stratification and biomarker discovery [81]. Recent advances have further increased the physiological relevance of PDOs through co-culture approaches incorporating immune cells and stromal components. Organoid-based immune co-culture systems have been shown to enable the study of tumor–immune interactions and immunotherapeutic responses, including modulation of the PD-1/PD-L1 axis [88]. In addition, organoid–lymphocyte co-cultures have demonstrated the capacity to generate tumor-reactive T cells from patient-derived material [89].

### 3.4. Microfluidic and Organ-on-Chip Systems

Microfluidic and OoC technologies have emerged as advanced in vitro platforms capable of recreating key aspects of the TME through the integration of 3D cell cultures within perfused microscale devices [90,91,92]. Unlike conventional static culture systems, these platforms provide precise control over fluid dynamics, oxygen availability, nutrient delivery, biochemical gradients, and mechanical forces, thereby reproducing dynamic physiological conditions that more closely resemble those found in vivo [93,94,95]. Generating interstitial flow through pressure gradients facilitates long-term tissue viability and enables the real-time monitoring of tumor behavior, making these microfluidic systems attractive tools for studying cancer biology and therapeutic responses [96]. In HNSCC, ex vivo perfusion models have successfully maintained tumor viability while preserving tissue architecture and cellular heterogeneity, enabling the functional assessment of chemotherapy and radiotherapy responses under physiologically relevant conditions [95]. More recently, Liao et al. developed a dual-channel microfluidic device using CAL-27-derived 3D cultures that generated stable concentration gradients for combinatorial drug screening [97]. This platform allowed for controlled drug exposure while maintaining spheroid integrity, illustrating the potential of microfluidic systems for preclinical therapeutic testing under dynamic perfusion conditions. Similarly, Bang et al. developed a vascularized tumor-on-chip platform integrating patient-derived HNSCC tumor spheroids with perfusable endothelial microchannels, enabling the reconstruction of key features of the TME under dynamic perfusion conditions [98]. This system enabled the investigation of tumor–vascular interactions while maintaining high spheroid viability and improved physiological relevance compared with conventional static cultures [98]. One of the major strengths of OoC technologies is their ability to integrate multiple cellular populations within spatially organized and dynamically perfused microenvironments, enabling the investigation of tumor–stroma interactions, paracrine signaling, and therapeutic responses [90,99]. The incorporation of PDOs into microfluidic platforms further enhances the translational potential of these systems by combining the biological complexity and inter-patient heterogeneity of organoids with controlled perfusion and microenvironmental regulation [90,100,101]. Despite their considerable potential, several technical challenges continue to limit the widespread implementation of microfluidic and OoC technologies. Device fabrication, protocol standardization, the long-term maintenance of multicellular cultures, and the incorporation of functional vascular and immune compartments remain technically demanding [90,102,103]. Moreover, most currently available OSCC microfluidic models rely on established cell lines rather than patient-derived tissues, limiting their ability to fully reproduce tumor heterogeneity [104,105].

### 3.5. Ex Vivo Tissue Models

Ex vivo tissue models, including patient-derived tumor explants and organotypic tumor slice cultures, represent an intermediate experimental platform between conventional in vitro models and in vivo studies in OSCC and HNSCC research [95,106]. These models are generated from freshly resected patient tumor specimens and maintained under short-term culture conditions, preserving native tissue architecture and relevant components of the TME, including ECM, stromal compartments, and resident non-malignant cell populations [95,107]. Unlike conventional monolayer cultures, explant-based models maintain spatial interactions between tumor cells and their surrounding microenvironment, enabling the investigation of tumor biology, therapeutic responses, and mechanisms of resistance in a more native context [107]. In HNSCC, patient-derived tumor slice cultures have demonstrated the feasibility of maintaining viable tumor tissue ex vivo while preserving histological architecture and cellular heterogeneity. For exemple, Gerlach et al. established an organotypic slice culture model from primary HNSCC surgical specimens, showing that the tumor slices remained viable during short-term culture and retained measurable responses to clinically relevant treatments, including CDDP, docetaxel, and cetuximab [108]. Treatment effects were evaluated through a histological assessment of tumor cell loss and apoptotic activation, including caspase-3 expression, demonstrating the potential of tumor slices as functional platforms for assessing individual therapeutic responses and investigating mechanisms associated with treatment resistance [108]. Further evidence supporting the translational relevance of ex vivo tumor models was provided by studies using primary HNSCC specimens to evaluate patient-specific responses to radiotherapy and chemotherapy. Capala et al. developed an ex vivo functional assay, in which freshly resected HNSCC tumor tissues were cultured and exposed to irradiation or CDDP treatment, demonstrating substantial inter-patient variability in treatment sensitivity [109]. These findings highlight the ability of ex vivo systems to capture tumor-specific biological differences that are not reproduced by established cell lines and support their potential application in personalized treatment stratification [109]. Beyond therapeutic testing, explant and organotypic slice models provide valuable systems for studying TME interactions. The preservation of extracellular matrix components, stromal cells, and spatial tumor organization enabled the investigation of processes such as invasion, stromal-mediated tumor progression, and microenvironment-driven resistance mechanisms [110]. Compared with dissociated cellular models, these platforms retain aspects of intratumoral heterogeneity, allowing for the evaluation of region-specific responses associated with differences in proliferation, cellular composition, and local tissue conditions [111]. Ex vivo models may also provide opportunities for immuno–oncology studies by maintaining resident immune populations for limited periods after tissue collection [112]. Although immune functionality progressively declines during culture due to the absence of vascular perfusion and systemic interactions, tumor slices allowed for the preliminary investigation of immune-related responses and treatment-induced changes within an intact tumor architecture [110,112]. Despite their physiological relevance, ex vivo explant models present several limitations, including restricted culture duration, variability between patient specimens, limited scalability, and challenges associated with protocol standardization [110,113,114]. The progressive loss of vascular perfusion and immune components also limits long-term applications [112].

A comparative summary of the main in vitro experimental platforms used in OSCC research, including their advantages and limitations, is presented in Table 1.

## 4. In Vivo Models Used to Study Oral Squamous Cell Carcinoma

In vivo models play a central role in OSCC research enabling the investigation of tumor growth, invasion, metastasis, and therapeutic response, within a systemic and physiologically environment [115]. In contrast to in vitro platforms, these models incorporate complex interactions between tumor cells and host-derived components, including immune populations, stromal cells, and vascular networks, thereby allowing for the study of tumor–host dynamics that cannot be fully reproduced ex vivo [116]. These models are particularly valuable for evaluating pharmacokinetics, drug toxicity, metastatic dissemination, and long-term therapeutic outcomes [115,117]. Furthermore, they enable the assessment of immune-mediated responses and microenvironment-driven resistance mechanisms, both of which are increasingly recognized as critical determinants of treatment failure in OSCC [42,116,118,119]. Despite their biological relevance, in vivo models are not without limitations. More recently, physiologically patient-derived xenograft (PDX) models have been integrated with high-resolution molecular profiling approaches, including whole-genome sequencing, transcriptomics, and 3D genome analysis, enabling the comprehensive characterization of tumor progression and therapy resistance [120]. These analyses identified 3D genome remodeling accompanied by the transcriptional changes associated with metabolic reprogramming during the acquisition of chemoresistance, and particularly involving mitochondrial energy metabolism [120]. Species-specific molecular differences, ethical concerns, high experimental costs, and intrinsic variability must all be considered when interpreting preclinical findings [121].

### 4.1. Xenograft and Humanized Mouse Models

Xenograft models represent one of the most widely used in vivo systems in OSCC research allowing implantation of human tumor cells into immunodeficient mice for the evaluation of tumor growth and therapeutic response [122]. Xenograft systems can be broadly classified into cell line-derived xenografts (CDXs) and PDXs. CDXs are established through implantation of immortalized cancer cell lines, offering high reproducibility and technical simplicity, whereas PDX models involve direct implantation of patient tumor fragments, thereby better preserving the intratumoral heterogeneity, stromal architecture, and molecular features of the original tumor [122,123]. These models provide a controlled platform for studying tumor biology within a living organism while preserving genetic alterations of the original cancer cells [122,123]. Subcutaneous xenografts generated by injecting tumor cells into the flank of immunodeficient mice are technically straightforward and highly reproducible [122,123]. Their accessibility facilitates the serial measurement of tumor volume, longitudinal monitoring of treatment response, and direct tissue collection, making them well-suited for early-stage drug evaluation, therapeutic susceptibility testing, and mechanistic studies [122,123]. Their main shortcoming, however, is the absence of any anatomical or microenvironmental context resembling the oral cavity, which limits their ability to model local invasion, lymphatic dissemination, and tissue-specific interactions [124,125]. Orthotopic xenograft models involve implantation of tumor cells directly into the anatomical site corresponding to the primary tumor, typically the tongue or oral mucosa [126,127]. This approach more faithfully recapitulates tumor–stroma interactions, vascularization patterns, and the metastatic behavior characteristic of OSCC including local invasion and cervical lymph node involvement in female mice [127,128]. He et al. demonstrated that orthotopically established OSCC PDX models preserve the pathological structure and molecular biological characteristics of the primary tumor [126]. Furthermore, the native tissue environment shapes metabolic gradients, hypoxia, and stromal signaling in ways that subcutaneous models fail to reproduce [126]. As demonstrated by Sheng et al., orthotopic implantation better preserves tissue-specific microenvironmental cues, making these models more informative for investigating response and resistance [129]. PDX models represent a further advancement over CDX systems by directly implanting tumor fragments obtained from surgical specimens, thereby preserving histological architecture, intratumoral heterogeneity, and patient-specific molecular diversity [126]. These features make PDX models particularly relevant for evaluating personalized therapeutic responses, validating predictive biomarkers, and investigating the mechanisms of acquired resistance [126,129]. However, their practical implementation is constrained by by considerable variability in engraftment rates, which depend on tumor-specific pathological features, including depth of invasion and nodal status [126,130]. Increasing evidence suggests that metabolic adaptations, particularly enhanced glycolysis, mitochondrial rewiring, and metabolic reprogramming contribute directly to tumor aggressiveness and reduced sensitivity to cytotoxic therapies in OSCC [61,131]. Highly glycolytic tumors appear more capable of adapting to therapeutic stress, thereby promoting tumor survival and therapeutic resistance [61,131]. Consequently, targeting tumor metabolism has emerged as a promising therapeutic strategy, reinforcing the value of xenograft and orthotopic models for investigating metabolic vulnerabilities and resistance mechanisms [132]. These include combinations involving dichloroacetate (DCA) and paclitaxel (PTX). Beyond serving as platforms for therapeutic testing, xenograft models have provided important insights into the metabolic reprogramming that underlies OSCC progression and therapeutic resistance. Their in vivo environment enables the investigation of tumor cell adaptation to fluctuations in oxygen and nutrient availability, revealing metabolic behaviours that cannot be fully reproduced in conventional in vitro cultures [133,134]. Although orthotopic and PDX studies specifically in OSCC remain limited, evidence from in vivo models of other solid tumors indicates that interactions with the native stromal compartment and vascular network can modulate glucose uptake and glycolytic flux, mitochondrial oxidative phosphorylation and metabolic flexibility, as well as resistance-associated adaptations such as enhanced antioxidant capacity, altered nutrient utilization, and metabolic states that support survival under therapeutic stress [133,134]. DCA, a metabolic modulator capable of shifting tumor metabolism toward oxidative phosphorylation through the inhibition of pyruvate dehydrogenase kinase, has shown the potential to enhance PTX efficacy by partially reversing metabolic resistance mechanisms and restoring chemosensitivity [135]. Beyond DCA, xenograft models have been used to evaluate the activity of other metabolic modulators in OSCC and head and neck cancers, including 2-deoxyglucose (2-DG), and mTOR and glutaminase inhibitors [132,136,137,138]. These models have also contributed to the investigation of ferroptosis-associated pathways and metabolic vulnerabilities in drug-tolerant persistent cells in HNSCC [119]. Although findings with DCA–PTX combinations are promising, evidence specifically in OSCC remains limited, as most available studies have been conducted in other solid tumor models [135]. Despite their utility, xenograft models present significant limitations in accurately modeling the human TME. A central constraint lies in the use of immunodeficient hosts, which lack functional adaptive immune responses, severely restricting the study of immune-mediated tumor surveillance, immune checkpoint dynamics, and immunotherapy efficacy, all of which are critical determinants of treatment outcomes in OSCC [42]. Stromal components within xenograft tumors are largely derived from the murine host; species-specific differences in cytokine signaling, extracellular matrix composition, and growth factor interactions may influence tumor behavior and drug response [139]. Consequently, the translational accuracy of these models may be limited, particularly for therapies targeting tumor–stroma interactions or immune-related pathways [140]. Metabolic interactions between tumor cells and their surrounding microenvironment also differ between xenograft systems and human tumors [139]. The establishment of hypoxic gradients, nutrient availability, and metabolic crosstalk may not fully replicate the complexity observed in native OSCC tissues [139]. As metabolic reprogramming plays a central role in therapy resistance and disease progression, incomplete reproduction of these conditions can affect the predictive value of preclinical findings [139]. Additionally, long-term in vitro expansion prior to CDX implantation can select for specific subclones, reducing the intratumoral diversity observed in primary tumors [42]. Traditional xenograft models have been widely used to evaluate tumor growth and drug response. However, their reliance on immunodeficient hosts represents a fundamental limitation when modeling immunotherapy [141,142]. Humanized mouse models were developed to partially overcome this limitation by incorporating components of the human immune system, including peripheral blood mononuclear cells (PBMC), hematopoietic stem cells (HSC), and bone marrow–liver–thymus reconstitution [143]. This approach enables the investigation of tumor–immune interactions and immune checkpoint dynamics in the context of oral cancer [143]. As highlighted by Khayatan et al., humanized models provide a more appropriate platform for evaluating immunotherapeutic strategies and understanding mechanisms of immune resistance in head and neck cancers [143]. These models have become particularly relevant for the preclinical evaluation of ICIs, including PD-1/PD-L1-targeted therapies, which currently play an increasingly important role in recurrent and metastatic OSCC management [144]. Nevertheless, despite overcoming the major limitation of conventional xenograft models, humanized mice present their own technical and biological challenges. Graft-versus-host disease represents a significant constraint in PBMC-based systems, while HSC-based reconstitution requires extended engraftment periods and remains incomplete relative to the full complexity of the human immune system [145]. In addition to their immunological relevance, these models may also provide important insights into the interplay between tumor metabolism and immune regulation [146,147].

### 4.2. Genetically Engineered Mouse Models

Genetically engineered mouse models (GEMMs) represent a powerful in vivo platform for studying OSCC development within an immunocompetent and physiologically intact host [137,148]. Unlike xenograft systems, GEMMs enable tumor initiation to occur in situ through tissue-specific activation or deletion of oncogenes and tumor suppressor genes [138,149]. Consequently, they more closely recapitulate the stepwise progression from epithelial dysplasia to invasive carcinoma and provide valuable insights into early tumorigenesis and disease evolution [137,148]. Several GEMMs have been developed to reproduce the molecular alterations characteristic of human OSCC and HNSCC, including conditional transformation-related protein 53 (*Trp53*) deletion, activation of *phosphatidylinositol-4,5-bisphosphate 3-kinase catalytic subunit alpha* (*PIK3CA*) signaling pathways, and epithelial-specific keratin-14 (*KRT14*)-driven oncogenic models [137,149,150,151]. The selective introduction of Trp53 deletion, alterations in PI3K/AKT/mTOR signaling, and the dysregulation of cell-cycle pathways enables a mechanistic investigation of how specific oncogenic drivers influence tumor progression, invasion, and metastatic dissemination [42]. Kalish et al. developed the KRTBmi-1 GEMM, in which *B cell-specific Moloney murine leukemia virus integration site 1* (*Bmi-1*) overexpression, a protein frequently elevated in human cancers and associated with CSC populations, is restricted to basal epithelial stem cells of the tongue, skin, and esophagus via a truncated human KRT14 promoter [137]. Using this model, the authors demonstrated that genetically induced Bmi-1 overexpression markedly increases susceptibility to chemically induced oral tumors while also altering critical metabolic pathways including oxidative phosphorylation and mTOR signaling, thereby increasing tumor aggressiveness [137]. Hamad et al. developed a complementary GEMM focusing on the role of the *nuclear factor erythroid 2-related factor 2* (*NRF2*), a transcription factor normally involved in cellular oxidative stress response, and demonstrated that *NRF2* can function as an oncogene in the context of oral carcinogenesis [148]. Importantly, unlike previous models based on *Kirsten rat sarcoma viral proto-oncogene* (*Kras*) mutations, which are rare in human oral cancer, this model more faithfully reflects the genetics of human HNSCC, where the loss of *p53* and *p16* occurs in more than 80% of cases [148]. In some models, genetically engineered backgrounds are combined with carcinogen exposure, most notably to 4-nitroquinoline-1-oxide (4-NQO), allowing for the study of gene–environment interactions in OSCC initiation and progression within an immunocompetent host [150]. GEMMs offer a unique opportunity with which to study tumor metabolism within an intact microenvironment. Unlike xenograft models, they enable the investigation of metabolic reprogramming throughout tumor initiation and progression, allowing for early metabolic events associated with carcinogenesis to be distinguished from adaptive metabolic changes that emerge during disease progression and therapeutic pressure [137,148,151]. Because tumors arise spontaneously in immunocompetent mice, GEMMs permit the study of metabolic interactions between cancer cells, stromal cells, and immune populations [131]. This provides valuable mechanistic insights into the relationship between genetic alterations, metabolic rewiring, and therapy resistance [131]. This is particularly relevant in OSCC, where metabolic reprogramming, including persistent alterations in glucose, mitochondrial, and lipid metabolism, together with adaptive metabolic plasticity, defined as the ability of tumor cells to dynamically adjust metabolic pathways in response to microenvironmental stress and therapeutic pressure, contribute significantly to therapy resistance [131]. GEMMs allow for the longitudinal evaluation of metabolic dependencies during tumor progression and under therapeutic pressure, enabling the dynamic tracking of metabolic shifts, including transitions between glycolytic and oxidative phenotypes, in response to treatment [137,151]. García-Carracedo et al. employed GEMMs harboring *PIK3CA* mutations to investigate the PI3K/AKT pathway, one of the principal regulatory axes of glucose metabolism in cancer, providing a mechanistic insight into how oncogenic PI3K activation drives metabolic reprogramming in oral epithelial cells [151]. Furthermore, the presence of a functional immune system enables the investigation of immunometabolic crosstalk, a dynamic and critical interface between tumor and immune cells [146,152]. As highlighted by Malvi et al., cancer evolution involves active metabolic competition for essential substrates such as glucose, amino acids, and lipids [153]. This competition, coupled with the release of immunosuppressive metabolites, such as lactate, creates metabolic barriers that drive immune evasion and ultimately determine the efficacy of the therapeutic response [153]. These characteristics make GEMMs particularly well-suited to integration with advanced molecular profiling approaches, including single-cell transcriptomics and other multi-omics tecnologies, enabling the correlation of genetic alterations with dynamic molecular changes throughout tumor evolution [154]. One of the principal advantages of GEMMs lies in their ability to model defined genetic drivers of OSCC within an immunologically intact host, enabling the study of tumor initiation, immune surveillance, and microenvironmental remodeling under conditions that more closely approximate human disease than xenograft-based systems [148,155]. Their immunocompetent background makes them particularly suited for preclinical evaluation of immunotherapeutic strategies, including ICIs and combination approaches pairing immunotherapy with metabolic or targeted agents. These models more faithfully reflect the tumor immune microenvironment of human OSCC [148,155]. Metabolic repurposing strategies, including the use of metformin as a mitochondrial complex I inhibitor and AMP-activated protein kinase activator, have also been proposed for evaluation in immunocompetent GEMM platforms, given their capacity to simultaneously modulate tumor metabolism and immune cell function [138]. Despite their biological relevance, GEMMs present important limitations. Murine oral epithelium differs histologically and molecularly from human oral mucosa, which may affect the generalizability of findings to human disease [149,150]. Furthermore, while GEMMs can faithfully model specific oncogenic drivers, they may incompletely capture the interpatient heterogeneity and complex mutational landscape characteristic of human OSCC, limiting their applicability for personalized therapeutic prediction [155]. Inducible models with oncogene overexpression or tumor suppressor knockout can provide more predictable tumorigenesis but also present challenges, including limited tissue specificity and the risk of transgene leakiness [155]. From a practical standpoint, GEMMs are associated with longer development times, higher costs, and variability in tumor latency and penetrance, which can introduce experimental variability and limit throughput [148,149].

### 4.3. Alternative Animal Models

Comparative oncology models, such as spontaneous canine oral squamous cell carcinoma (COSCC) in dogs, offer a particularly valuable translational framework [156]. Unlike experimentally induced tumors in rodents, canine oral cancers arise naturally in immunocompetent animals and evolve within a complex physiological environment that closely resembles human disease [156]. These models, therefore, provide an opportunity with which to study tumor biology, immune interactions, and therapeutic responses under conditions that more closely reflect clinical foundations [156]. A study by Guscetti et al. provides a comprehensive characterization of these similarities through comparative transcriptomic profiling of COSCC and human HNSCC [156]. The analysis demonstrated strong transcriptional concordance, particularly in pathways related to cell-cycle progression, EMT, and oncogenic signaling networks, including E2F transcription factor family (*E2F*), *MYC*, *KRAS*, mTORC1, and TGF-β [156]. Canine tumors display a loss of epithelial differentiation and marked overexpression of KRT14 and KRT17, molecular features commonly observed in HNSCC [156]. Notably, a partial EMT program driven by zinc finger E-box binding homeobox 2 (*ZEB2*) was identified, accompanied by overexpression of immune checkpoint molecules PD-L1 and cytotoxic T-lymphocyte-associated protein (CTLA)-4, suggesting the presence of an immunosuppressive TME closely resembling that of HNSCC [156]. Conserved therapeutic vulnerabilities were also identified between canine and human tumors, including a shared dependence on the Cyclin-Dependent Kinases 4 and 6 and *E2F* (*CDK4/6*–*E2F*) axis [156]. Pharmacological inhibition of CDK4/6 using agents, such as palbociclib, demonstrated high efficacy in COSCC-derived cell lines, reinforcing the translational potential of this model for preclinical drug evaluation [156]. Beyond cell-cycle dependencies, the conserved oncogenic signaling networks identified in COSCC, particularly mTORC1, *KRAS*, and *MYC*, are well-established drivers of metabolic reprogramming in human cancers [157,158,159]. Their activation promotes enhanced glycolysis, glutamine dependency, and metabolic flexibility, enabling tumor cells to switch between glycolytic metabolism and oxidative phosphorylation according to microenvironmental conditions and therapeutic stress [157,158,159]. The translational relevance of canine models extends beyond biological similarity. Companion animals share environmental exposures, genetic diversity, and disease progression patterns that are often more representative of human cancers than artificially induced laboratory models, making comparative oncology settings particularity informative for both veterinary and human oncology [156,160]. Despite their translational relevance, studies involving companion animals require strict ethical standards, including informed owner consent and the prioritization of animal welfare [161,162]. Ethical constraints and the limited availability of standardized tumor samples remain significant limitations of this model [161,162]. In addition to mammalian systems, the zebrafish (*Danio rerio*) has emerged as an increasingly significant platform in oncology research [163]. While rodent models are limited by their anatomical opacity and the small size of the oral cavity, the development of the transparent adult zebrafish casper strain has substantially advanced in vivo transplantation analysis [163]. This model is particularly suited for investigating OSCC metabolism, as its optical transparency enables high-resolution, real-time imaging of metabolic fluxes and nutrient uptake within the TME [163]. This unique imaging capability enables researchers to investigate how metabolic alterations evolve during tumor progression and how they relate to invasion, angiogenesis, and therapeutic response, providing insights that are difficult to obtain in conventional mammalian models [163,164]. Unlike canine or murine models, wherein tissue depth and opacity require sophisticated imaging approaches to visualize tumor progression, zebrafish xenografts provide a unique platform for the direct observation of OSCC progression. Furthermore, they enable the investigation of the metabolic reprogramming and glycolytic activity associated with therapeutic resistance [143,156]. Wen et al. demonstrated that zebrafish xenografts represent an effective and cost-efficient model for investigating how specific molecular alterations influence tumor growth, invasion, and therapeutic response through real-time imaging approaches [163]. In this system, inhibition of MMP9 affected invasion, vascularization, and systemic dissemination of oral cancer cells [163]. Metsäniitty et al. employed zebrafish larvae to investigate the impact of extracellular vesicles derived from oral bacteria *A. actinomycetemcomitans* and *P. gingivalis* on OSCC growth and metastasis [164]. The study demonstrated that this platform enables rapid and cost-effective monitoring of how external factors influence tumor progression and is particularly well suited for assessing metastatic dissemination [164]. Nevertheless, the zebrafish model presents important limitations. Compounds with hydrophobic properties may not be readily absorbed through the embryonic medium, necessitating more complex delivery methods such as microinjection or cell pre-treatment [164]. During the first 30 days of development, larvae possess only innate immune cells and lack a functional adaptive immune response [164]. While this facilitates xenotransplantation without rejection, it limits the study of immune-dependent therapeutic mechanisms [164]. Furthermore, for certain processes, such as angiogenesis, the underlying molecular mechanisms in zebrafish remain incompletely understood and require further investigation [163]. The Syrian golden hamster (*Mesocricetus auratus*) represents one of the most established animal models for the study of oral carcinogenesis, particularly for investigating chemoprevention and therapeutic efficacy [165,166]. Oral carcinogenesis is induced in the hamster buccal pouch (HBP) through the topical application of chemical carcinogens, such as 7,12-dimethylbenz[a]anthracene, generating tumors that share histological, morphological, biochemical, and molecular characteristics with human oral malignancies [165,166]. This model enables observation of the complete disease sequence from hyperplasia and dysplasia to well-differentiated invasive carcinoma, faithfully replicating the multistep progression observed in humans [166]. The HBP model has proven particularly valuable for evaluating the toxicity of treatments, including radiotoxicity and oral mucositis in peri-tumoral precancerous tissue, which functions as the dose-limiting tissue [165,166]. It also supports longitudinal follow-up (for example, over 28 days post treatment) to assess tumor response, remission, and tissue recovery in a non-invasive manner [165,166]. In addition, *Mesocricetus auratus* is considered an ideal model for studying experimental oral cancer chemoprevention because chemically induced cancers in this system closely resemble human oral neoplasms at the biological level [165,166]. Consequently, this model provides a robust platform for evaluating the efficacy of novel therapeutic agents in reversing pre-neoplastic histological alterations [165,166]. However, this model also presents limitations. Therapeutic response is size-dependent. While small and medium tumors achieved complete remission rates of 73 and 42% respectively, only 13% of large tumors reached complete remission, mirroring the clinical difficulty of treating bulky and unresectable disease [160,165,166]. Additionally, the HBP lacks an anatomical counterpart in humans, which may affect the direct extrapolation of some findings to human oral cavity tumors [166]. Looking ahead, the field appears to be moving towards hybrid and complementary experimental strategies [167,168,169,170,171,172]. These approaches integrate the mechanistic depth of GEMMs, the translational fidelity of PDX and comparative oncology models, and the metabolic imaging capabilities of zebrafish. Their continued development is also supported by a broader commitment to the 3Rs framework and the progressive integration of advanced non-animal platforms [167,168,169,170,171,172].

A comparative summary of the main in vivo experimental models used in OSCC research, highlighting their principal applications, advantages, and limitations, is provided in Table 2.

## 5. Integrating Experimental Models with Multi-Omics Approaches

Recent advances in OSCC research have increasingly combined the experimental models with multi-omics technologies to improve the characterization of tumor heterogeneity, metabolic reprogramming, TME interactions, and therapeutic responses [173]. Rather than replacing experimental platforms, multi-omics approaches complement 2D cultures, organoids, OoC systems, explants, PDXs, and GEMMs. By integrating genomics, transcriptomics, metabolomics, spatial transcriptomics, and single-cell sequencing, they provide high-resolution molecular information that cannot be obtained through morphological or functional analyses alone [173]. Transcriptomic analyses performed in patient samples and experimental models, particularly in organoids and PDXs, have identified molecular pathways involved in tumor progression, EMT, stromal remodeling, and immune modulation, providing important reference datasets for experimental model validation [42]. More recently, spatial transcriptomics has advanced the study of intratumoral heterogeneity by assessing the spatial organization of gene expression within tumors, revealing distinct metabolic and genetic adaptations between hypoxic tumor cores and invasive fronts [131,173,174]. Using GEMM, multi-omics demonstrated that miR-181a-5p regulates peroxisome proliferator-activated receptor-driven lipid metabolism by decreasing the expression of perilipin 2 and fatty acid-binding protein 4, while the loss of *miR-181a-5p* increases lipid metabolism, leading to membrane biosynthesis and metastasis [175]. Spatial transcriptomic analyses performed in human OSCC tissues found that epithelial cells, B cells, plasma cells, mast cells, and dendritic cells were more abundant in OSCC than in the normal group, while endothelial cells, fibroblasts, T cells, and natural killer cells were more prevalent in the normal group [176]. The authors also revealed different functional fibroblast and endothelial cell subtypes with differing gene expression and markers that seem to be associated with tumor progression. For example, some OSCC fibroblast cells were shown to be regulated in a similar way to normal fibroblasts, while some endothelial cells were more invasive than others. Interestingly, these normal-like fibroblasts showed the highest interaction intensity with the invasive epithelial cells. The release of insulin-like growth factor 1 (IGF1) ligands by the normal-like fibroblasts may lead to the inhibition of Forkhead Box O1 (FOXO1) through the insulin-like growth factor 1 receptor (IGF1/IGF1R) axis [176]. However, invasive epithelial cells upregulate integrin subunit (ITG) A6 and ITGB4, to which IGF1 can also bind, reducing oxidative stress [176]. Additionally, spatial and single-cell transcriptomics analysis identified hypometabolic, normal, and hypermetabolic regions, with the hypermetabolic area being located in the epithelial region [177]. In this region, lactate generated by epithelial cells is taken up by fibroblasts, driving their transition into inflammatory CAFs [177]. These inflammatory CAFs present higher expression of *HIF1A*, which enhances C-X-C motif chemokine (CXC) ligand 12 production and secretion [177]. Increased secretion of CXC ligand 12 subsequently promotes the recruitment of regulatory T cells, leading to higher TGF-β levels in the TME and contributing to the establishment of an immunosuppressive microenvironment [177]. Moreover, epithelial cells characterized by elevated glutathione levels exhibited stronger interactions with endothelial cells and were predominantly localized within central tumor regions and invasive fronts [178]. Furthermore, glutathione *S*-transferase omega 2 (GSTO2)-high epithelial cells demonstrated increased communication with immune cell populations and were enriched in areas with greater immune cell infiltration [178]. These cells also showed the activation of metabolic pathways associated with enhanced energy production and detoxification processes, in addition to interactions with interferon-stimulated genes and immunoproteasome subunits [178]. In contrast, epithelial cells with low glutathione or GSTO2 expression displayed a more dispersed distribution throughout the tumor tissue [178]. This type of analysis was also used to differentiate molecular subtypes of OSCC and cell populations associated with different survival outcomes in immunotherapy treatment [179,180]. Enrichment of myogenic factor 5^+^ muscle satellite cells correlated with mono-immunotherapy non-responders, while p-selectin^+^ high endothelial venules and apolipoprotein D^+^ myofibroblastic CAFs were associated with better outcomes [180]. In addition, it was found that low expression of CD147 in the tumor invasive front was correlated with better clinical outcome, while actin-related protein 2/3 complex subunit 4 upregulation was associated with poor prognosis [181]. Dysregulated tryptophan metabolism was found to promote macrophage phenotype reprograming to a complement component 1, q subcomponent, C chain^+^ phenotype, inducing OSCC progression and correlating with a worse prognosis [182]. Another study described three distinct OSCC regions, a tumor core, a leading edge, and a transitory region [183]. To this end, a higher tumor core gene signature was shown to correlate with better overall survival (OS), disease-specific survival (DSS), and progression-free survival (PFS), while a high leading-edge gene signature was associated with worse DSS [183]. Moreover, it was also found that young and old tongue squamous cell carcinoma patients showed different transcriptomic and molecular features. For example, mitogen-activated protein kinase and JAK-STAT pathway enrichment was found in young patients compared to older ones [184]. A different study divided OSCC into two molecular distinct subtypes termed CMS1 and CMS2, which correlated with the chromobox protein homolog 3 (CBX3):interleukin 1 receptor antagonist (IL1RN) ratio [185]. The CMS1 had a higher CBX3-to-IL1RN ratio and was correlated with worse outcomes [185]. A transcriptomic analysis integrating machine learning and single-cell and spatial data identified low *S*-adenosyl-methionine and SH3 domain-containing protein 1 (SASH1) expression in HNSCC tumors; further analysis found a correlation between low SASH1 expression and poor survival [186]. Furthermore, high expression of SASH1 was associated with lower sensitivity to ZM447439, an Aurora kinase inhibitor, and KU-55933, an ataxia-telangiectasia (AT)-mutated kinase inhibitor, as well as with a higher sensitivity to AZD7762, a Chk1/2 inhibitor, and AZD6738, an AT and Rad3-related kinase inhibitor [186]. In parallel, PDOs from HNSCC and OSCC faithfully reproduced genetic alterations, invasive behavior, and patient-specific therapeutic responses, making them valuable platforms for linking multi-omics profiles with drug sensitivity and resistance [81,84,187]. For instance, patient-derived head and neck organoids were shown to retain histopathological and molecular features and DNA alterations, such as the copy-number variations and gene mutations (*TP53*, *NOTCH1*, *PIK3CA*, *FAT1*, and *APOB*), observed in head and neck cancer patients [81]. Nonetheless, the sample size was limited, which might have prevented statistically significative correlations between organoid response and patient response to radiotherapy or chemoradiotherapy. However, other factors, such as unattainability of surgery, could be in play, since it is usually performed in HNSCC patients and cannot be accounted for in organoid models and the lack of TME in the organoid models tested. Regarding biomarkers analysis, organoids showed that *PIK3CA* mutation was not related to alpelisib response, corroborating the disappointing results in clinical trials [81]. However, loss of *CDKN2A* was shown to predict sensitivity to protein arginine methyltransferase 5 inhibitors and should be further investigated [81]. A different study with a 3D pillar-and-well array culture system using patient-derived HNSCC cells showed that the radioresponse group correlated with patients presenting less adverse clinicopathological features and a lower recurrence rate [188]. In addition, when compared to 2D models, 3D models demonstrated higher CSC-associated marker expression and mostly decreased levels of EMT-related genes while showing higher resistance to CDDP and cetuximab [189]. A 3D bioprinted model was also shown to be more resistant than 3D spheroids to CDDP, reflecting a more faithful patient response [190]. Recently, a human patient-derived OSCC organoid biobank was created; transcriptomic analysis identified CUB domain-containing protein 1 (CDCP1) as a potential contributor to CDDP resistance, potentially through the promotion of Wnt/β-catenin-driven stemness [191]. In addition, depletion of CDCP1 using nanoparticles overcame CDDP resistance in PDX models [191]. Clinically, OSCC patients presented with dysregulation of glycerophospholipid and sphingolipid metabolism, while the gene diacylglycerol kinase gamma (DGKG) promoted cell migration and invasion through lipid metabolism reprogramming, highlighting its potential as a therapeutic target [192]. Strikingly, high-invasive and low-invasive OSCC cells elicited different metabolic features. For instance, high-invasive cells showed increased glycine and arachidonic acid metabolism, de novo fatty acid synthesis, and omega-3 polyunsaturated fatty acid release compared to low-invasive cells [62]. However, different models underscored differences in metabolism. For example, 3D models using tongue cancer cells demonstrated enhanced basal and compensatory glycolytic activity, characterized by increased levels of glycolytic intermediates, together with enhanced mitochondrial metabolism, biomass production, and amino acid biosynthesis, compared with 2D models. In contrast, xenografts exhibited lower levels of glycolytic intermediates compared with 3D models, while maintaining similar mitochondrial metabolism, amino acid biosynthesis, and biomass production [193]. Interestingly, the study suggests that cells in 2D models lose their characteristic metabolism, maintaining a more similar intercellular metabolism than that observed in 3D models or xenografts [193]. 3D models under hypoxic conditions have been used widely in OSCC research, as hypoxia promotes glucose metabolism reprogramming and provides a relevant platform for assessing the therapeutic response [194]. The 3D microenvironment of OSCC spheroids was found to promote human epidermal growth factor receptor 3 expression, which inhances cell survival and proliferation while conferring resistance to EGFR and human EGFR2 inhibitors through HIF-1α modulation [194]. In HNSCC organoids, *TP53* wild-type models responded to nutlin-3, a mouse double minute 2 inhibitor that represses p53 wild-type cell growth, while sensitivity to CDDP, carboplatin, and cetuximab varied among patient-derived lines [84]. CDDP showed greater efficacy than carboplatin and demonstrated a synergistic effect with radiotherapy, suggesting that it may be the preferred option for radiosensitized HNSCC patients. On the other hand, the results with cetuximab corroborate the data from recent studies that point to no correlation between EGFR expression or increased gene copy number and cetuximab response [84]. Moreover, cetuximab produced only an additive effect when combined with radiotherapy. Importantly, organoid responses to radiotherapy correlated with patient outcomes, although the sample analyzed was small [84]. Variable responses were also observed for targeted therapies with some differences associated with specific genetic alterations. This was the case with alpelisib and vemurafenib, a B-Raf proto-oncogene (*BRAF*) inhibitor, whereas the variable responses observed with niraparib, a poly(ADP-ribose) polymerase inhibitor, everolimus, an mTOR inhibitor, and AZD4547, a fibroblast growth factor receptor inhibitor, could not be explained by the genetic mutations evaluated [84]. Furthermore, comparative RNA-seq of spontaneous canine OSCC demonstrated strong molecular similarity to human HNSCC, such as *ZEB2*-driven partial EMT and upregulation of *KRT14* and *KRT17*, supporting its relevance as an alternative in vivo model [156]. The conservation of therapeutic vulnerabilities was also observed, including overexpression of PD-L1, CTLA-4, and CDK4/6, highlighting the value of comparative oncology for validating clinically relevant targets [156]. Collectively, these studies demonstrate that integrating multi-omics technologies with complementary experimental platforms provides a more comprehensive understanding of OSCC biology than either approach alone. While organoids and 3D cultures enable patient-specific transcriptomic, metabolomic, and drug-response profiling, PDXs and GEMMs facilitate the validation of molecular mechanisms in physiologically relevant in vivo settings [101,195]. Likewise, spatial transcriptomics and single-cell analyses performed in clinical specimens preserve tissue architecture and cellular interactions, allowing for the identification of metabolic niches and therapeutic vulnerabilities [196]. Together, these complementary strategies substantially strengthen the translational relevance of OSCC research.

## 6. Nanosystems Applied to Oral Squamous Cell Carcinoma

**Figure 2:**
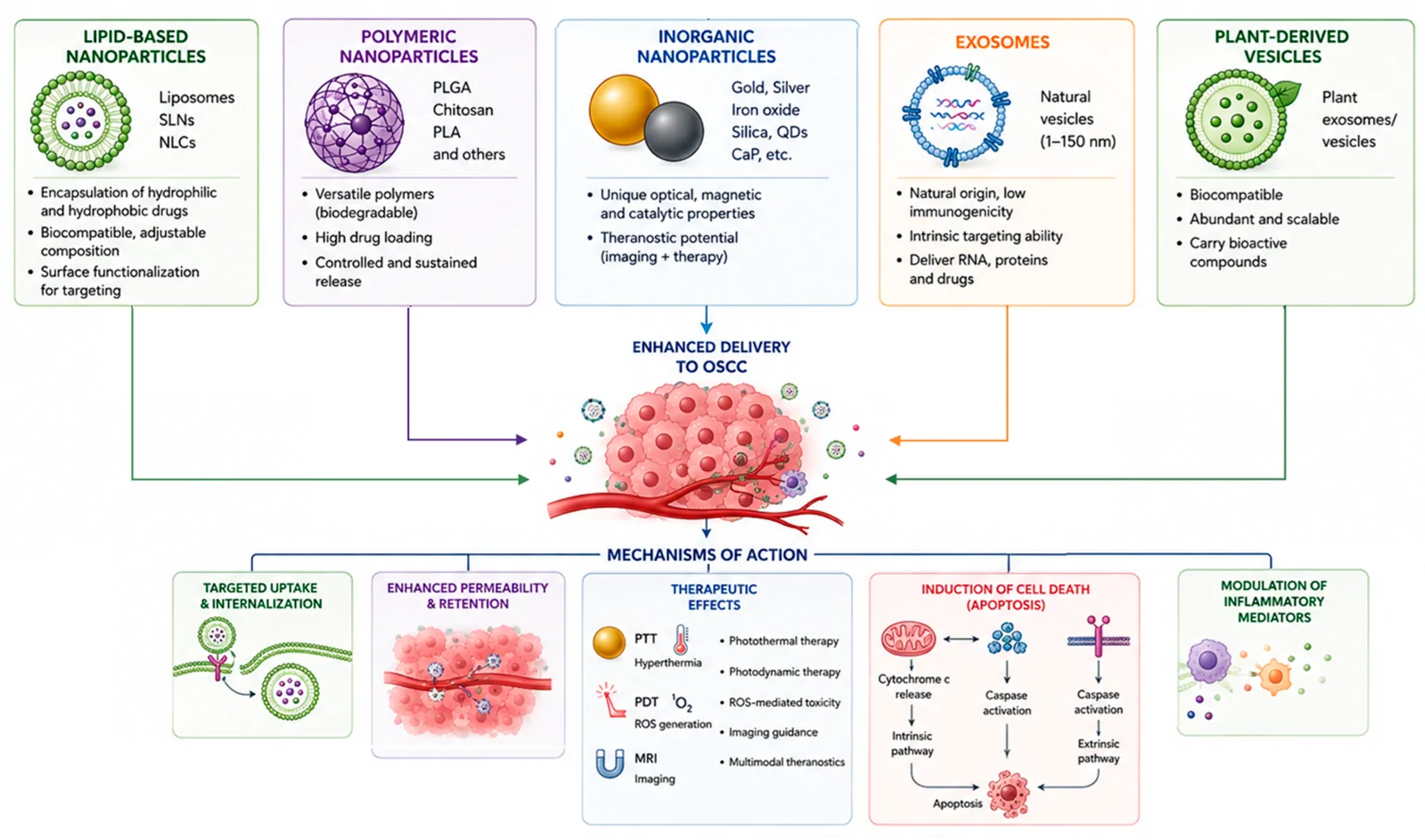
Nanotechnology-based delivery platforms and therapeutic mechanisms in oral squamous cell carcinoma. Different nanocarrier systems, including lipid-based nanoparticles, polymeric nanoparticles, inorganic nanoparticles, exosomes, and plant-derived vesicles, can improve the delivery, tumour accumulation, and therapeutic efficacy of anticancer agents in OSCC. These nanoplatforms facilitate targeted uptake, enhanced intracellular delivery, controlled drug release, and therapeutic effects through mechanisms such as photothermal therapy (PTT), photodynamic therapy (PDT), reactive oxygen species (ROS) generation, imaging-guided therapy, and multimodal theranostic approaches. Nanocarrier-mediated anticancer activity may involve induction of apoptotic pathways, including mitochondrial cytochrome c release, caspase activation, and modulation of inflammatory mediators within the tumour microenvironment. Abbreviations: OSCC, oral squamous cell carcinoma; SLNs, solid lipid nanoparticles; NLCs, nanostructured lipid carriers; PLGA, poly(lactic-co-glycolic acid); PLA, polylactic acid; QDs, quantum dots; CaP, calcium phosphate; PTT, photothermal therapy; PDT, photodynamic therapy; ROS, reactive oxygen species; MRI, magnetic resonance imaging.

Nanosystems have been widely investigated to improve drug efficacy by increasing penetration and retention, and to reduce toxicity by specifically targeting cancer cells [197]. Several nanosystems, including solid lipid nanoparticles, liposomes, polymeric micelles, metallic nanoparticles, and dendrimes, have been created and assessed for the treatment of several types of cancer [197]. Some of the nanocarriers used in OSCC include lipid nanoparticles, polymeric nanoparticles, inorganic nanoparticles, exosomes, and plant-derived vesicles [198,199] (Figure 2). Lipid nanoparticles comprise a diverse class of drug delivery systems that include liposomes, solid lipid nanoparticles, and nanostructured lipid carriers (NLCs) [198,199]. Liposomes are phospholipid vesicles composed of one or more lipid bilayers surrounding an aqueous core, which enables the encapsulation of both hydrophilic and lipophilic compounds, making them versatile drug delivery systems [198,199]. In addition to their excellent biocompatibility, liposomes have the capacity for self-assembly and can be readily functionalized with targeting ligands [198,199]. On the other hand, their capacity to load hydrophobic drugs is limited by the available bilayer volume and they are not entirely immunologically inert [198,199]. Solid lipid nanoparticles (SLNs) exhibit high physical stability, favorable biocompatibility, enhanced bioavailability, and controlled drug release [198,200]. They are also amenable to large-scale production, demonstrate high batch-to-batch reproducibility, and can be prepared without the use of toxic organic solvents [198,200]. Due to their small size and large surface area, SLNs can be readily functionalized to improve delivery efficiency [198,200]. They can be administered via multiple routes, including oral delivery in the form of aqueous dispersions, capsules, and other dosage forms [198,200]. However, they exhibit low drug-loading efficiency due to their highly ordered crystalline structure; drug leakage may occur during storage as a result of recrystallization processes [198,200]. NLCs are lipid-based drug delivery systems composed of a solid lipid matrix at room temperature and characterized by favorable biocompatibility [198,200]. Compared with other lipid nanocarriers, they offer improved physical stability, more controlled release profiles, and higher drug-loading capacity, largely due to their imperfect crystalline structure, which reduces drug expulsion by limiting lipid recrystallization during manufacturing and storage [198,200]. NLCs were developed through the modification of SLNs by partially replacing solid with liquid lipids, resulting in a mixed lipid matrix with enhanced drug incorporation and controlled release properties [198,200]. Nonetheless, a key limitation of NLCs is their relatively limited capacity for surface functionalization compared with other nanocarrier systems [198,200]. Polymeric nanoparticles can be formed using a great range of polymers, such as poly(lactide-co-glycolide) (PLGA), chitosan, and poly(D,L-lactic acid) (PLA), which are usually biocompatible, biodegradable, and nontoxic [201]. Polymeric nanoparticles have a high surface-to-volume ratio, reproducibility, nonimmunogenicity, ease of synthesis and characterization, stability upon administration, and enhanced absorption properties with low cost [201]. However, polymeric nanoparticles can present toxicity due to toxic monomer aggregation and toxic degradation products [201]. Nonetheless, toxicity is associated with quantum size and the polymeric materials, with natural materials having less toxicity than synthetic ones [201]. The addition of some stabilizers can also increase their toxicity [201]. Inorganic nanoparticles have unique physicochemical properties and demonstrate the potential for theranostic applications integrating diagnostic and therapeutic functions within a single platform [202,203]. Achieving this level of multifunctionality is often more challenging with lipid-based or polymeric nanoparticles [202,203]. Furthermore, inorganic nanomaterials generally possess well-defined and predictable characteristics including size, shape, and surface properties, wich facilitate their optimization for biomedical applications [202,203]. Common examples include gold and silver nanoparticles, metal oxides such as silica and iron oxide nanoparticles, quantum dots, and calcium phosphate-based nanoparticles [202,203]. Among these, gold nanoparticles are the most extensively studied due to their high chemical and physical stability, biocompatibility, ease of synthesis, and readily modifiable surface [204]. Their tunable optical and thermal properties make them particularly attractive for photothermal therapy, in which near-infrared (NIR) irradiation is converted into localized heat to induce tumor cell death, and photodynamic therapy, which combines a photosensitizing agent, light, and oxygen to generate ROS, which induce cell death [204]. Despite these advantages, the toxicity of these nanoparticles can be influenced by factors such as size, shape, surface chemistry, and cellular uptake mechanisms [204]. Silver nanoparticles can be tailored through variations in size, shape, morphology, and surface characteristics [205]. In addition, silver is an abundant and relatively low-cost material; however, silver nanoparticle clinical translation has been limited by stability issues, particularly due to their susceptibility to oxidation in biological environments [205]. Iron oxide nanoparticles are easily synthesized and versatile nanomaterials, with unique magnetic properties combined with favorable biocompatibility [206,207]. Moreover, iron oxide nanoparticle-based theranostic systems allow for the tracking of nanoparticle distribution, the monitoring of therapeutic responses, and the evaluation of treatment efficacy [206,207]. Following surface modification, superparamagnetic iron oxide nanoparticles can be used in a wide range of biomedical applications, including the targeted delivery of therapeutic agents, biosensing, and tissue repair [206,207]. Titanium dioxide is a commonly used nanomaterial that was shown to not be cytotoxic, not stimulate hormetic growth at lower concentrations, and does not affect chemotherapeutic agents anticancer effects; however, it can act as a photosensitizer [208,209]. Although its toxicity is considered to be low, this depends on the size and crystal form [210]. Titanium oxide nanoparticles can form agglomerates, while modifications can improve their stability and prevent their agglomeration [210]. Mesoporous silica nanoparticles possess versatile physicochemical properties including scalable and cost-effective synthesis, hydrophilicity, favorable biocompatibility, large surface area, high pore volume, and controllable particle size [211]. These characteristics make them attractive drug delivery systems capable of controlled drug loading and release [211]. Quantum dots are semiconductor nanomaterials with exceptional optical properties typically composed of a heavy-metal core coated with a semiconductor shell [212]. Their nanoscale size, tunable composition, high quantum yield, and size-dependent emission make them attractive for biomedical imaging [212]. However, their optical performance is highly sensitive to physicochemical changes, which can lead to aggregation, degradation, and instability [212]. Quantum dots produced via organometallic synthesis often show limited aqueous solubility; large-scale production is challenging due to the need for extensive surface modifications to maintain consistent properties [212]. Impurities may further compromise their optical behavior [212]. Moreover, they lack intrinsic targeting specificity and have been associated with potential cytotoxicity [212]. Depending on their size and composition, they may be rapidly cleared via renal filtration or accumulate in tissues, raising concerns about long-term safety [212]. Exosomes are small extracellular membrane-bound vesicles released by most cell types [213]. Due to their cellular origin, they show low immunogenicity, high biocompatibility, and an inherent capacity to traverse biological barriers including cell membranes and the blood–brain barrier [213]. Nevertheless, their clinical translation is hindered by several limitations such as challenges in large-scale production and isolation, instability during storage, rapid systemic clearance, and poor control over drug loading efficiency [213]. Plant-derived vesicles are characterized by abundant natural sources, low production costs, and high yield [214]. They do not carry zoonotic or human pathogens, resulting in a reduced risk of immune reactions in vivo compared with mammalian-derived vesicles, and are capable of resisting digestive enzymes [214]. In addition, they exhibit several advantageous properties, including favorable biocompatibility, low toxicity, structural rigidity, and the ability to cross biological barriers, as well as improved circulation stability [214]. Nevertheless, their molecular composition can vary significantly depending on the plant species and tissue of origin, which may influence their functional properties [214]. To overcome some of the limitations associated with nanosystems, various modifications can be implemented. These include the incorporation of additional biomaterials, targeting ligands, proteins, or enzymes to enhance tumor specificity and improve nanoparticle penetration within the TME [215]. However, such modifications may also introduce undesirable effects, including slower drug release or reduced drug-load efficiency, potentially reducing their cytotoxicity [215]. Furthermore, enzymes are susceptible to rapid degradation in vivo; their conjugation to nanoparticles may impair their activity through conformational changes or obstruction of the active site [215].

### Preclinical and Clinical Studies Using Nanoparticles

**Figure 3:**
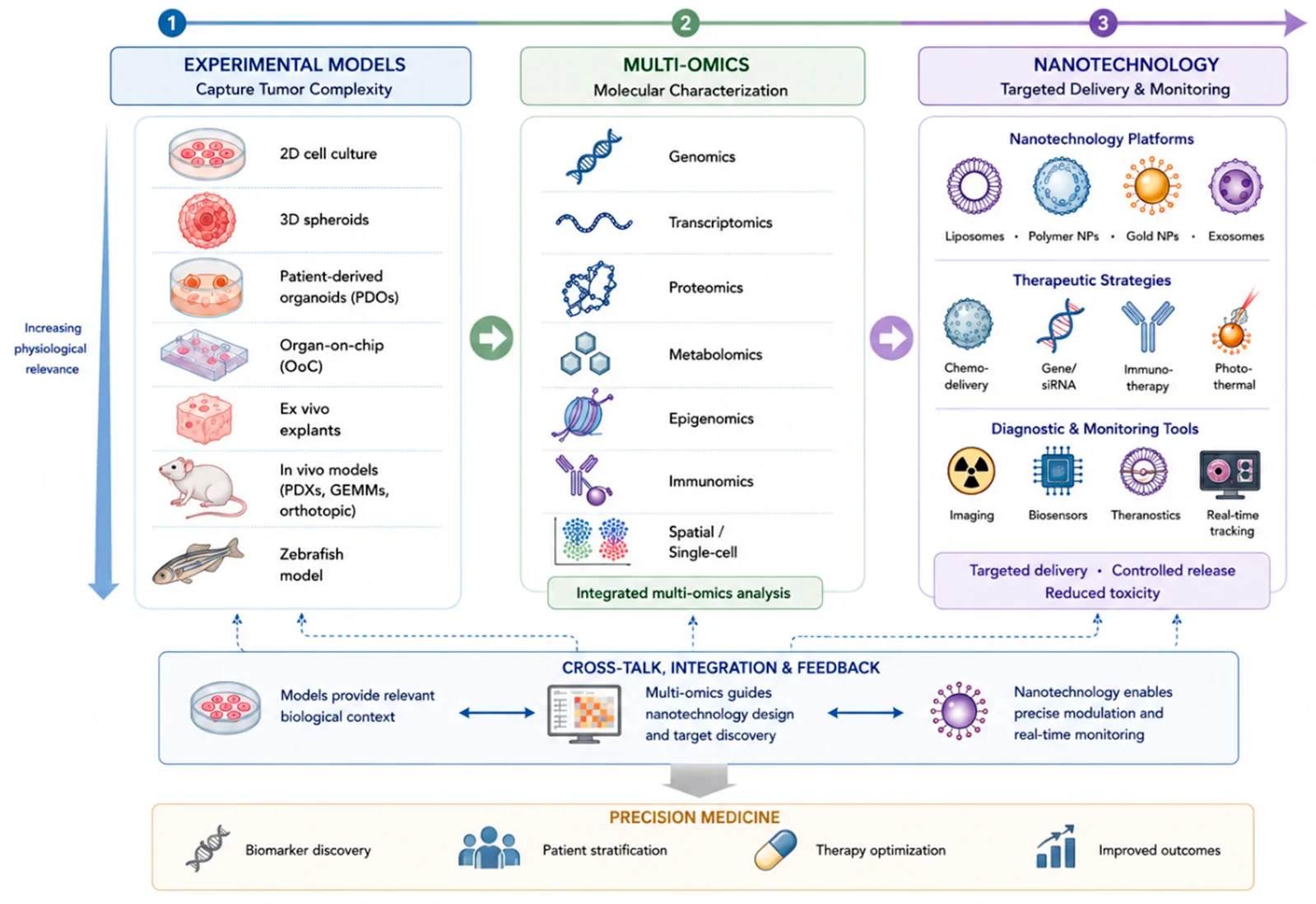
Integration of experimental models, multi-omics technologies, and nanotechnology within the translational research pipeline of OSCC. Together, these complementary approaches provide a comprehensive framework for biomarker discovery, therapeutic target identification, and the development of precision medicine strategies. Experimental models provide complementary biological platforms to investigate tumor complexity while multi-omics analyses identify molecular alterations and therapeutic vulnerabilities. These findings guide the development of nanotechnology-based diagnostic and therapeutic strategies establishing an iterative feedback process that accelerates biomarker discovery, target validation, and precision medicine. This figure was created using a generative AI tool based on an author-provided conceptual description and subsequently reviewed and edited by the authors.

The study of nanoparticles combines in vitro and in vivo models to assess pharmacokinetic parameters and nanoformulation efficacy. For instance, in vitro studies are commonly performed to evaluate drug release under physiologically relevant conditions, cellular uptake, the selective uptake of functionalized nanoparticles by target cancer cells, and their anticancer effects [216,217]. In OSCC, calcium carbonate (CaCO_3_)-based nanoparticles containing protamine sulfate were found to enhance the efficiency of miR-200c plasmid DNA transfection and to decrease migration and viability in vitro and tumor growth in vivo [218,219]. CaCO_3_ is a mineral biomaterial naturally present in the human body, typically in the form of nanocrystals, and is characterized by high biocompatibility and biodegradability [218,219]. In this nanoparticle formulation, protamine sulfate was incorporated to enhance the transfection efficiency of CaCO_3_-mediated plasmid DNA delivery [218,219]. A folic acid-conjugated solid lipid nanoparticle loaded with PTX or ascorbic acid exhibited an initial burst, followed by a slow and sustained release, resulting in prolonged drug availability in the plasma [220,221]. In vivo, the combination of PTX- and ascorbic acid-loaded nanoparticles demonstrated greater therapeutic efficacy than either nanoparticle formulation administered alone [220,221]. Folic acid is essential for DNA base synthesis; many cancer cells exhibit increased expression of folate receptors. Therefore, the functionalization of nanoparticles with folic acid can promote preferential binding to folate receptor-expressing tumor cells, potentially enhancing nanoparticle accumulation and therapeutic efficacy [220,221]. Glutathione-sensitive and folate-targeted nanoparticles loaded with PTX were also assessed in OSCC [222]. Folate targeting improved cellular uptake in vitro and biodistribution in vivo, leading to increased nanopartticle accumulation within the tumor [222]. The system also exhibited favorable biocompatibility [222]. Furthermore, the glutathione-responsive design enabled controlled PTX release following drug encapsulation within the nanoparticle carrier [222]. Nonetheless, despite lower cytotoxicity than free PTX in vitro, the nanoparticles effectively reduced tumor growth in vivo [222]. Similarly, cathepsin B is overexpressed in several cancer types; thus, cathepsin B-responsible nanoparticles were used to load capivasertib, an AKT inhibitor, to reduce off-target accumulation [223]. These nanoparticles showed a similar effect to free capivasertib in 2D and 3D models, while they were also able to radiosensitize tumor cells in vitro and in orthotopic xenograft mice [224]. With an identical design, polyethylenimine (PEI)-modified Fe_3_O_4_ nanoparticles were conjugated with DNA to create a magnetic nanovector capable of specifically targeting cancer cells [225]. PEI was used to coat the DNA from degradation [225]. These nanoparticles were then used to deliver the pACTERT-TNF-related apoptosis-inducing ligand (TRAIL) plasmid to encode TRAIL to tumor cells, resulting in enhanced TRAIL-mediated cytotoxicity in both in vitro and in vivo models [225]. Doxorubicin–methotrexate-loaded nanoparticles have also been tested and were shown to significantly reduce vascular endothelial growth factor-C and *MMP-2* mRNA expression both through oral and intravenous administration, which seems to be related to their anticancer effects [226,227]. An analysis of human OSCC samples showed that ubiquitin-specific protease 30 (USP30) was overexpressed in OSCC, leading to increased cell viability and glutamine consumption while repressing apoptosis [228]. Zeolitic imidazolate framework-8 (ZIF-8)–polydopamine (PDA)–polyethylene glycol-thioketal (PEGTK) nanoparticles containing the USP30 inhibitor MF-094 led to higher cytotoxicity and glutamine consumption reduction in vitro and increased reduction in tumor growth in vivo compared to the free drug [228]. ZIF-8 is a metal–organic framework material; its nanoparticles were found to be relatively stable under physiological conditions but prone to degradation under acidic conditions [228,229]. PDA forms stable coatings and presents high biocompatibility and metal adsorption capacity, allowing for its wide use in nanoformulations [230]. The PEGTK coating enables the nanoparticles to acquire an ROS-responsive trait [228,231]. Dipalmitoyl phosphatidic acid (DPPA) nanoparticles loaded with the ERK1/2 inhibitor MK-8353 were evaluated in comparison with PLGA nanoparticles containing the same inhibitor [232]. DPPA nanoparticles showed stronger repression of proliferation and clonogenic capacity, and, additionally, exerted antiangiogenic effects [232]. In both orthotopic xenograft and PDX models, DPPA nanoparticles enhanced tumor growth inhibition compared to the blank nanoparticles, free MK-8353, and PLGA-based nanoparticles loaded with MK-8353 [232]. DPPA is a phospholipid whose nanoparticles are effective in encapsulating and releasing drugs while also presenting inherent angiogenic and antitumoral effects [233]. PLGA is a polymer with favorable biocompatibility and biodegradability that is widely used in nanocarrier systems [234]. Moreover, PLGA nanoparticles containing the natural product toosendanin showed an efficient release profile while enhancing cytotoxicity compared to the free compound [234]. In CDX and PDX models, these nanoparticles also led to increased tumor growth reduction [234]. PLGA nanoparticles containing gold or silver and bioactive quinacrine showed higher cytotoxicity than nanoquinacrine or PLGA-based gold or silver nanoparticles [235,236]. Moreover, the nanoparticles containing gold and quinacrine reduced the expression of pro-inflammatory cytokines (IL-6, IL-1β, IL-8, and TNF-α) and CSC markers (CD44 and CD133) in H-357 cells, spheroids, and in a post-EMT variant of this cell line [235,236]. Nonetheless, the nanoparticles containing gold and quinacrine were more effective at reducing IL-6 and IL-1β in cells positive for both p53 and p21 [235,236]. Similarly, in the H-357 post-EMT variant, the nanoparticles containing gold and quinacrine increased ROS and nitric oxide and reduced angiogenic markers more effectively in p53 and p21 positive cells [235,236]. In a fertilized egg, nanoparticles containing gold and quinacrine decreased angiogenesis in a p53- and p21-dependent manner, while the silver nanoparticles decreased angiogenesis; however, the p53 and p21 statuses were not explored [235,236]. Furthermore, the nanoparticles containing gold and quinacrine reduced tumor growth more effectively in vivo than nanoquinacrine or PLGA-based gold nanoparticles [235,236]. It has been suggested that gold- and quinacrine-containing nanoparticles exert their anticancer effects at least in part by not only inducing DNA damage and re-replication stress but also by reducing the expression of base excision repair and DNA replication fork-related proteins [237]. The combination of the gold-containing nanoparticles, which function as a photothermal agent by converting NIR radiation into heat, and quinacrine with NIR significantly improved their cytotoxicity [238]. The addition of TRAIL further enhanced this effect in vitro, which may be partly explained by TRAIL-mediated inhibition of heat shock protein 70 (HSP70), thereby contributing to the observed antitumor activity [238]. The triple combination also reduced tumor growth in vivo [238]. The comparison of both gold and silver nanoparticles containing quinacrine showed that the gold nanoparticles led to lower inhibition concentration 50 (IC_50_) in oral cancer cell lines while arresting cells at S phase, promoting DNA damage and inhibiting topoisomerase more effectively than the silver nanoparticles [239]. In another study, pH-responsive PLGA nanoparticles (PLGA-Dlink) coated with polyethylene glycol (PEG), which prolongs nanoparticle systemic circulation and reduces immunogenicity, were used to deliver a small interfering RNA targeting LINC02613 [240,241]. This approach effectively reduced cell viability in vitro and inhibited tumor growth in both CDX and PDX models [241]. However, PEG has been reported to induce hypersensitivity reactions and is considered non-biodegradable [242]. pH-sensitive piperlongumine-loaded PLGA nanoparticles containing sodium bicarbonate were examined in OSCC [243,244,245]. Sodium bicarbonate was incorporated to regulate the pH of the TME and potentially induce cell death through the release of high amounts of Na^+^. The nanoparticles also contained D-α-tocopherol poly(ethylene glycol) 1000 succinate, used as an emulsifier with potential P-glycoprotein inhibitory activity, and polyvinyl alcohol, which was included as a stabilizer [243,244,245]. These nanoparticles showed increased drug release and cellular uptake under acidic conditions. Zebrafish were used to assess nanoparticle circulation, since they are transparent and present a robust blood circulation [245]. Approximately 30% of the injected nanoparticles were still circulating even after 72 h. These nanoparticles were also observable in mice blood plasma, even after 48 h, while accumulating in high levels at the tumor sites [245]. Moreover, the nanoparticles resensitized oral cancer cells to CDDP more effectively than free piperlongumine [245]. Poly-γ-benzyl-*L*-glutamate, a biodegradable polymer with favorable mechanical strength and thermal stability, was combined with PEG to synthetize nanoparticles loaded with the herpes simplex virus thymidine kinase gene [246,247]. These nanoparticles exhibited higher gene transfer efficiency and enhanced cytotoxicity in both OSCC cells and a golden hamster model [246,247]. Macrophage membrane-encapsulated poly(methyl vinyl ether-alt-maleic anhydride)–phenylboronic acid–doxorubicin (MM@PMVEMA–PBA–DOX) nanoparticles were synthesized to exploit the ability of macrophages to communicate with cancer cells, thereby increasing their accumulation in OSCC tumors compared with nanoparticles lacking the macrophage membrane coating [216]. In addition, the macrophage membrane enabled the nanoparticles to evade immune recognition, thereby reducing immune clearance and prolonging their circulation time [216]. PMVEMA is a polyanhydride, a non-cytotoxic and non-mutagenic surface-eroding polymer that can degrade into carboxylic acids with lower acidity than those produced by polyesters, that is used in nanoparticle synthesis and presents strong bioadhesive interactions with components of the intestinal mucosa [248]. PBA was also incorporated in the nanosystem, since it forms a boronate ester bond with doxorubicin that is hydrolyzed under low pH levels, allowing for a pH-sensitive release of the drug [216]. In OSCC, MM@PMVEMA–PBA–DOX showed similar effects to PMVEMA–PBA–DOX nanoparticles both in vitro and in vivo, but with more increased cytotoxicity than free doxorubicin [216]. Although MM@PMVEMA–PBA–DOX nanoparticles demonstrated improved targeting in OSCC tumors, the presence of the macrophage membrane affected drug release [216]. Lipid nanoparticles coated with PD-1–overexpressing macrophage membrane loaded with inhibitor of protein phosphatase 2A siRNA were constructed to overcome the poor siRNA stability in circulation and to improve cellular uptake and nanoparticle accumulation within the tumor [249]. These nanoparticles showed increased cytotoxicity and migration inhibition in vitro while being more effective in inhibiting tumor growth in vivo [249]. A reduction-responsive nanoparticle loaded with siRNA targeting lipocalin 2, which is involved in metastasis and EGFR resistance, was constructed by mixing the amphiphilic polymer methoxy PEG-disulfide-PLGA with the cationic lipid G0C14 and tested in OSCC [250]. In the presence of glutathione, the nanoparticles quickly dissolved, liberating the siRNA and inducing cell death of oral cancer cell lines [250]. In vivo experiments showed that these nanoparticles accumulated more effectively in the tumor than free siRNA and exhibited a prolonged circulation time [250]. These nanoparticles also reduced tumor volume more effectively in both xenograft and PDX models [250]. Fisetin-loaded cross-linked zein nanoparticles containing fucoidan and hyaluronic acid were tested in OSCC cells [251]. Hyaluronic acid and fucoidan were used to stabilize the nanoparticles and promote active targeting to the tumor and the surrounding TME cells. Zein, a natural protein, was shown to contribute to passive tumor targeting [251]. These nanoparticles showed an initial burst release followed by a slow and continuous release, while leading to slightly enhanced cell death when compared with Fisetin-loaded cross-linked zein nanoparticles containing only hyaluronic acid or fucoidan [251]. In vivo studies showed that the nanoparticles were safe and reduced the espression of markers associated with tumor cells, CSC, and tumor endothelial cells, while promoting apoptosis compared to the control group and the fisetin suspension [251]. Genistein-loaded lactalbumin nanoparticles showed increased solubility and controlled drug release [252]. Lactalbumin can promote synthesis of antioxidants and has shown antiproliferative effects in several cancer cell types [252]. The nanoparticles showed increased cytotoxicity in the JHU011 cell line compared to free genistein [252]. The nanoparticles inhibited mitogen-activated protein kinase-activated protein kinase 3, which is directly involved in the transcriptional regulation of enhancer of zeste homolog 2, thereby reducing cell proliferation and invasion while inducing apoptosis [252]. Consistently, in vivo studies showed improved biodistribution, aqueous dispersibility, and biocompatibility [252]. Hyaluronic acid nanoparticles encapsulating combretastatin A4 phosphate demonstrated high encapsulation efficiency and a favorable drug-release profile [253]. These nanoparticles exhibited greater cytotoxicity than the free drug in an OSCC cell line, while showing no increase in cytotoxicity toward a normal epithelial cell line [253]. In addition, the nanoparticle formulation was more effective at inhibiting tumor growth in vivo and prolonged the systemic circulation of combretastatin A4 phosphate, maintaining higher drug concentrations in the bloodstream for an extended period [253]. Gelatin nanoparticles loaded with the photosensitizer indocyanine green and NSC74859 were shown to be degraded by MMPs, which are upregulated in OSCC [254]. The nanoparticles exhibited high stability and a favorable safety profile, and achieved high drug-loading efficiency together with a controlled drug-release profile [254]. Combining these nanoparticles with laser irradiation increased apoptosis considerably in vitro [254]. Similarly, a combination of laser irradiation and nanoparticles loaded with indocyanine green and NSC74859 was the most effective in reducing tumor volume and immunosupressive cells from the TME [254]. Nucleic acid analogues can be used to create drug carriers, since they exhibit favorable biocompatibility and thermal stability [255]. However, they show weak chemical and enzymatic stability in vivo and their production is costly, time-consuming, and complex [255]. However, a study using bidentate Janus-type thymidine-adenosine L-ribonucleoside showed that it could form monomeric self-assembled nucleoside nanoparticles with low cytotoxicity in vitro and in vivo [255]. These nanoparticles are easier to produce, and are biodegradable, when compared to other commonly used systems [255]. In OSCC, these nanoparticles, loaded with 5-fluorouracil (5-FU), led to the increased stability of 5-FU in blood circulation and uptake in tumor tissue [255]. Moreover, the nanoparticles led to a greater reduction in tumor growth in vivo than with free 5-FU [255]. Metal–organic framework nanoparticles offer several advantages as drug delivery systems, including a highly tunable architecture, favorable biocompatibility, and biodegradability [256]. Nevertheless, their tumor-targeting efficiency remains limited, which may restrict their therapeutic performance [256]. Metal–organic framework nanoparticles were coated with dental pulp mesenchymal stem cell membranes containing CXC receptor 2 [256]. Since OSCC cells secrete CXC ligand 8, CXC receptor 2-functionalized nanoparticles are attracted to the tumor, as evidenced by their increased uptake by oral cancer cells compared with unmodified metal–organic framework nanoparticles [256]. In OSCC, the nanoparticles coated with dental pulp mesenchymal stem cell membranes and containing doxorubicin led to increased apoptosis and tumor growth reduction in vivo [256]. Copper sulfide nanoparticles have also been explored in oral cancer, owing to their high mesoporous drug loading and photo-activated drug release [257]. The GE11 peptide, which binds to EGFR without triggering receptor activation, was incorporated into copper sulfide nanoparticles to improve their limited tumor-targeting capability [257]. These nanoparticles, loaded with galangin and combined with NIR, led to similar toxicity, cell migration, and invasion inhibition to free galangin in oral cancer cells [257]. In mice, GE11 incorporation increased the presence of the nanoparticles in the OSCC tumor while showing greater tumor reduction even compared with free galangin [257]. The effect of gold nanoparticles has been explored in CAFs, since it seems that most nanoparticles that reach the tumor site are retained in the extracellular matrix or uptaken by non-cancer cells [202,258]. Several nanoparticle sizes were used; it was found that the smallest particles (3 nm) were the most effective in reducing CAF migration by decreasing the expression of N-cadherin, vimentin, α-smooth muscle actin, and fibroblast-specific protein 1 [259]. These proteins are associated with the active states of CAFs, which are more likely to migrate when in an active state [259]. The 3 nm nanoparticles were also uptaken in increased quantity, which might explain these effects [259]. Moreover, the communication between CAFs and OSCC cells was hindered after treatment with the 3 nm nanoparticles, reducing OSCC cell viability and migration [259]. Using SCC9 cells plus CAFs in a xenograft model, the 3 nm nanoparticles led to the greatest reduction in tumor volume out of all the nanoparticle sizes explored [259]. Gold nanoparticles coated with phosphorylated focal adhesion kinase antibody were also tested in combination with no-ozone cold plasma that was developed for safe use in the mouth [259]. The direct treatment led to higher cell death than the plasma-activated media, followed by the addition of cells [259]. Moreover, the concurrent treatment led to enhanced reduction in tumor volume in vivo than the gold nanoparticles or the cold plasma therapy alone [259]. Hypoxia can be an obstacle to photodynamic therapy (PDT), since most treatments are based on the production of singlet oxygen [208]. To overcome this issue, metal polypyridyl complexes, such as the Ru(II) complex, TLD-1433, have been investigated [208]. TLD-1433 was shown to cause DNA damage and has phototoxic effects under hypoxia [208]. Titanium dioxide coupled with a Ru complex with a structure similar to the TLD1433 nanoparticles incorporating HIF-1α siRNA showed increased cytotoxicity in the presence of light in the HN6 cell line, while it led to worse IC_50_ in the HSC-6 cell line both under normoxia and hypoxia [208]. In a PDX model, the nanoparticles plus irradiation showed the most significant tumor volume reduction, while it was the most effective treatment in reducing oral carcinogenesis in a 4-NQO mice model [208]. A nanoparticle constituted with phospholipid dioleoylphosphatidic acid coated in 1,2-dioleoyl-3-trimethylammonium-propane (chloride salt) was used to trap CDDP [260]. 1,2-dioctadecanoyl-*sn*-glycero-3-phosphoehanolamine conjugated with aminoethyl anisamide was incoporated in the outer membrane to target the nanoparticles to cancer cells, since it binds to sigma receptors that are usually overexpressed [260]. These nanoparticles led to lower IC_50_ than free CDDP in vitro, while PDT using photosan-2 as the photosensitizer, followed by nanoparticle treatment, led to the greatest reduction in tumor volume in vivo [260]. Human serum albumin-based nanoparticles loaded with indocyanine green, a photosensitizer, and CDDP plus NIR led to increased cytotoxicity in a CAL-27 cells and CAFs, compared to HaCaT cells and normal fibroblasts, while reducing tumor growth in vivo [261]. Moreover, the presence of the nanoparticles was increased in the tumor site compared to free indocyanine green and CDDP [261]. The advantage of albumin-based nanoparticles is that albumin is internalized into cells through macropinocytosis, which has been shown to be promoted by hyperactivated EGFR, rat sarcoma, and PIK3CA signaling, which is commonly observed in HNSCC [262]. In addition, some nanoparticles are able to modulate immune cells. For instance, Prussian blue nanoparticles can lead to the conversion of M2-polarized tumor-associated macrophage into the M1 macrophage type, which has tumor-suppressive activity [263]. In OSCC, this type of nanoparticle showed high biocompatibility while leading to macrophage polarization from M2 to M1 in vitro and in vivo [263]. Ultra-small Prussian blue nanoparticles were also incorporated into mesoporous calcium–silicate nanoparticles to produce an acid-responsive iron-based nanocomposite [264]. Iron can induce chemodynamic therapy (CDT) reactions [264]. CDT agents convert H_2_O_2_ into ROS in the TME through Fenton or Fenton-like reactions, leading to apoptosis induction [264]. The nanoparticle system was shown to be cytotoxic against oral cancer cells but not normal cell lines, while reducing cell migration and invasion more effectively than the other treatments explored [265]. It seems that the nanoparticles decreased the expression of glutathione peroxidase 4 (GPX4) and the cysteine/glutamate antiporter xCT, inducing ferroptosis [265]. In vivo, the nanosystem led to significant tumor growth reduction [265]. NLCs containing piperine and quercetin exhibited enhanced cellular uptake and were more effective than the free compounds in reducing oral cancer cell proliferation while also demonstrating a satisfactory biodistribution profile [266]. Quantum dots serve as ideal contrast agents for fluorescence-guided surgery, due to their brightness and stability [267]. Cadmium selenide/cadmium sulfide/zinc sulfide quantum dots coated with sulfobetaine polymers and conjugated to A20FMDV2 peptide, which targets αVβ6 integrin that is overexpressed in OSCC, accumulated in both 2D and 3D models, but with lower levels in spheroids at a concentration of 50 nM [267]. Moreover, quantum dots accumulated mostly at the periphery of the spheroids [267]. Silver sulfide quantum dots bonded to podoplanin monoclonal antibodies were evaluated as probes and for the targeting of partial EMT in OSCC, and showed a favorable biosafety profile [268]. Podoplanin is a cancer marker of partial EMT; the incorporated antibody allows for the targeting of cells expressing this marker, while silver sulfide allows for phototherapy in the presence of NIR [268]. The quantum dot system plus NIR led to increased cytotoxicity in vitro, while in vivo, the quantum dots bonded to monoclonal antibodies accumulated more efficiently in the tumor [268]. In vivo, the addition of NIR also led to greater tumor volume reduction, while this system also overcame resistance to PD-1 immunotherapy [268]. Nanocatalysts coated with hyaluronic acid, cancer cell membranes, and manganese were constructed to take advantage of the camouflage conferred by the cancer cell membrane, increasing their presence in the tumor site and the manganese’s potential to induce CDT reactions [264]. The nanocatalysts coated with manganese showed increased uptake and cytotoxicity in cancer cells compared to normal cells while leading to reduced tumor volume in vivo [264]. Nanoscale forms of some compounds have been synthetized and explored in cancer treatment [269]. For instance, hydroxyapatite is a calcium-based biomaterial with bioactivity, osteoconductivity, and favorable biocompatibility that can serve as a drug carrier. It can be synthetized as nanohydroxypatite [269]. TME-responsive nanohydroxypatite was more cytotoxic than its non-responsive counterpart in vitro [269]. When combined with cold atmospheric plasma, it showed a synergystic effect, leading to higher cell inhibition than the separate treatments. Similarly, the combinatorial approach reduced in vivo tumor growth more effectively [269]. Supramolecular nanodrugs also present high bioavailability, since they are capable of undergoing absorptive endocytosis and are interesting options for cancer treatment [270]. For example, LEE011-FFERGD/CDDP, a supramolecular nanodrug containing ribociclib (LEE011), a CDK4/6 inhibitor, overcame resistance to CDDP both in vitro and in vivo by repressing B-cell lymphoma 2 associated athanogene 1 [270]. FFERGD is a peptide that self-assembles into ordered supramolecular structures, allowing for the incorporation of drugs [270]. Plant-derived nanovesicles have also been explored because they exhibit favorable biocompatibility, inherent bioactivity, and potential anticancer effects [271]. Several natural nanovesicles were tested in vitro, with most showing inhibitory effects regarding cell proliferation and wound healing [271]. Of these natural vesicles, Panax notoginseng-derived nanovesicles were the most effective, leading to a reduction in the primary tumor burden and cervical lymph node metastasis in vivo [271]. Moreover, Panax notoginseng-derived nanovesicles exhibited efficient and progressive cellular uptake and a favorable safety profile, and accumulated at the tumor site [271]. It was found that these nanovesicles induced ferroptosis, PANoptosis, and ROS–autophagy [271]. Bitter melon-derived extracellular vesicles also led to cell viability reduction by inducing apoptosis through ROS production and increased JUN protein expression [272]. Double-layered membrane vesicles (DMVs) extracted from attenuated *Porphyromonas gingivalis* and incorporating poly(β-amino ester) nanoparticles loaded with the photosensitizer IR780 were also evaluated in OSCC [272]. Furthermore, since bitter melon-derived vesicles reduced the expression of NLR family pyrin domain containing 3 (NLRP3), which is associated with 5-FU resistance, their combination with 5-FU was investigated. This combination, both in vitro and in vivo, produced greater antitumor effects than either 5-FU or bitter melon-derived vesicles alone [272]. The attenuation of *Porphyromonas gingivalis* was performed using lysozyme to reduce the content, lipopolysaccharide, which is the main source of DMV-associated toxicity [273]. Moreover, the nanoparticles containing IR780 improved photostability; when combined with laser irradiation, this approach increased cytotoxicity in vitro [273]. The DMVs did not further improve the anticancer effect [273]. Nonetheless, the DMV formulation led to the greatest reduction in tumor volume in vivo [273]. Exosomes derived from the stem cells of human deciduous exfoliated teeth were found to inhibit the growth, migration, and tube formation of endothelial cells while promoting cell death [274]. The exosomes contain miR-100-5p and miR-1246, which target vascular endothelial growth factor A [274]. Using fertilized chicken eggs, exosomes were also shown to inhibit angiogenesis, while in mice, they significantly reduced tumor volume [274]. Tumor exosome-based nanoparticles containing indocyanine green and gefitinib, an EGFR tyrosine kinase inhibitor, showed the sustained release of gefitinib but reached an accumulative release ratio of 92.9% after 10 min of irradiation [275]. Irradiation also promoted cellular uptake [275]. The presence in the tumor was increased for the exosomes compared to the free drugs [275]. In vivo, it was shown that the exosomes exerted higher phototermal effects, while irradiating the exosome vesicles considerably increased ROS production and decreased tumor volume [275]. Hydrogels are platforms with a 3D porous network that possess a high drug-loading capacity, allowing for sustained and controlled drug release and excellent biocompatibility [276]. Due to their tissue-adhesion properties, these platforms allow for the prolonged permanence of the carried drugs, which increases bioavailability and minimizes off-target toxicity [276]. Several hydrogel-based systems have been explored in OSCCs. For instance, a celecoxib and γ-aminobutyric acid-loaded wheat gluten–sodium alginate nanocarrier hydrogel showed high drug-loading efficiency, constant drug release, and lower IC_50_ with the increased concentration of celecoxib [277]. Additionally, the injectable hydrogel led to a reduction in tumor volume and in the number of tumor lesions in vivo [277]. Wheat gluten is a natural low-cost protein that can be used as a gelling agent but shows poor mechanical proprieties [277]. Sodium alginate is a softer biopolymer that presents pH sensitivity, which is advantageous for drug release at a physiological pH; it was incorporated to improve the mechanical properties of the wheat gluten hydrogel [277]. A hydrogel based on carboxymethyl chitosan and four-arm PEG-benzaldehyde was developed to incorporate single-atom nanozymes, which promote CDT-induced cell death [276]. The nanozymes were modified with PEI to facilitate the surface adsorption of glucose oxidase, thereby enhancing H_2_O_2_ generation, which is essential for CDT-induced apoptosis [276]. The hydrogel also incorporated liposomes containing buthionine sulfoximine, a γ-glutamylcysteine synthetase inhibitor, which induces ferroptosis by inhibiting glutathione synthesis and decreasing GPX4 expression [276]. This system showed initial burst release followed by a continuous and gradual liberation [276]. The nanozymes combined with the liposomes produced a greater reduction in cell viability than either treatment alone. In vivo, the hydrogel containing both the nanozymes and the liposomes also reduced tumor growth more effectively than PEI-modified iron single-atom nanozymes, either with or without glucose oxidase, or PEI-modified iron single-atom nanozymes combined with liposome-encapsulated buthionine sulfoximine [276]. In addition, a lipid carrier nanostructured using ovucire as the lipid encapsulating pitavastatin–*Pinus densiflora* oil was assessed in the gingival carcinoma HGF-1 cell line, eliciting high entrapment efficiency and constant and prolonged drug release [278]. Several formulations were then tested; the hydrogel containing the optimized nanostructured lipid carrier showed the highest cytotoxicity in vitro [278]. A polyplex hydrogel consisting of DNA and PEG was loaded with zinc oxide nanoparticles containing CDDP [279]. The DNA contained a tumor-specific promoter responsive to a pH of 6.5 and sequences designed to target the anti-apoptotic protein Bcl-xL, while zinc oxide nanoparticles were incorporated due to their potential to promote ROS production [279]. This nanohydrogel exhibited high drug-loading capacity, sustained drug release, and antibacterial activity, while reducing cell viability and migration in vitro [279]. In vivo, enhanced tumor accumulation was observed; the formulation containing zinc oxide nanoparticles and CDDP achieved greater tumor growth inhibition and improved survival compared with the other tested treatments [279]. Furthermore, a sodium alginate hydrogel incorporated upconversion NaYF_4_ nanoparticles, which convert NIR to the wavelength that activates these photosensitizers [280]. These nanoparticles were coated with mesoporous silica and folic acid and loaded with amphiphilic chlorin e6. The hydrogel also contained magnesium particles [280]. Chlorin e6 is a photosensitizer used in PDT [280]. The hydrogel combined with NIR led to the most effective cytotoxic effect compared to NIR or the hydrogel alone, while exhibiting favorable biocompatibility and promoting osteogenic differentiation due to the presence of the magnesium particles [280]. Similar results were observed for in vivo experiments, where the hydrogel plus NIR led to the highest reduction in tumor volume and led to osteogenic promotion [280]. Some nanosystems have demonstrated similar or even lower cytotoxicity than free drugs in cancer cell lines, while exhibiting superior anticancer effects in vivo [281]. This discrepancy likely reflects the greater importance of tumor targeting under in vivo conditions than in conventional in vitro 2D models [281]. While free drugs may exhibit limited tumor accumulation following systemic administration, nanoparticles are often specifically designed to target tumor tissues, thereby increasing effective local drug concentrations and enhancing therapeutic efficacy [281]. Although 3D models are interesting alternatives, their production is usually time-consuming and associated with low yield or is complicated and difficult to implement in the laboratory [281]. To address these challenges, an agarose-based microwell microsystem was developed to enable the rapid and reproducible production of large numbers of spheroids through a single pipetting step [281]. Although this system was initially validated using colon cancer cells, it could potentially be adapted for oral cancer cell lines [281]. Notably, cellular uptake was found to be greater in 3D spheroids than in 2D cultures, further emphasizing the limitations of conventional monolayer models for evaluating nanoparticle behavior [281]. In addition, nanoparticle size is a critical determinant of antitumor activity, as it influences circulation half-life, biodistribution, tumor targeting, cellular uptake, and tumor penetration [282]. For instance, smaller nanoparticles may be internalized more efficiently by cancer cells and tumor-associated cells, leading to enhanced cytotoxicity [259]. Although nanocarrier systems have been extensively investigated, particularly in recent years, no encapsulated drug has yet been approved for the treatment of HNSCC [283]. Nevertheless, recent studies have shown that nab-PTX combined with nivolumab in patients with PD-1 inhibitor-refractory recurrent metastatic HNSCC resulted in improved objective response rates and PFS compared with those reported for standard therapy [283]. Moreover, in p16-positive oropharyngeal squamous cell carcinoma, 2-year DSS and OS were significantly improved with a nab-PTX-based induction chemotherapy regimen (nab-PTX, CDDP, and 5-FU) compared with a docetaxel-based regimen (docetaxel, CDDP, and 5-FU), with no significant increase in grade 3 or 4 adverse events [284]. A phase I trial explored induction with carboplatin and nab-PTX followed by concomitant 5-FU, hydroxyurea, and twice-daily radiation therapy administered every other week plus nab-PTX in previously treated HNSCC [285]. This therapy modality achieved a PFS of 60 months and OS rates of 23 and 25%, respectively, comparable to pembrolizumab plus chemotherapy [286]. Notably, patients who responded to induction therapy experienced substantially improved outcomes with a PFS of 5 years and OS rates of 38 and 44%, respectively, suggesting that this treatment strategy may be particularly beneficial in selected patients [286]. Similarly, nab-PTX combined with cetuximab and carboplatin as a first-line therapy for recurrent or metastatic HNSCC resulted in favorable clinical outcomes, including a median PFS of 6.1 months, an objective response rate (ORR) of 60%, a disease control rate of 89%, and a median OS of 17.8 months [262]. While PFS was comparable to that reported for the EXTREME regimen and to PTX or docetaxel given with cetuximab and a platinum agent, both OS and ORR were superior [262]. Furthermore, nab-PTX and CDDP, followed by concurrent chemoradiotherapy in patients with locally advanced nasopharyngeal carcinoma, led to a 3-year PFS of 86.1% and a cancer-specific survival of 91.7%, with manageable toxicity [287]. Percutaneous transcatheter intra-arterial administration of nab-PTX was shown to be feasible in advanced oral cavity, oropharyngeal, and hypopharyngeal squamous cell carcinomas, achieving an ORR of 75%, with a low incidence of complications [288,289]. In patients with locally advanced HNSCC, induction therapy with nab-PTX and CDDP followed by CDDP-based chemoradiotherapy was compared with induction nab-PTX followed by cetuximab-based radiotherapy [285]. The CDDP-containing regimen achieved a substantially higher complete response rate than the cetuximab-containing regimen (70 vs. 20%, respectively) [285]. Furthermore, among patients with HPV-positive oropharyngeal squamous cell carcinoma, the CDDP-based approach resulted in 2-year locoregional control (LRC), PFS, and OS rates of 100, 90, and 100%, respectively, compared with 72, 54, and 67% for the cetuximab-based regimen [285]. Similarly, in patients with HPV-negative HNSCC, the CDDP-containing regimen achieved 2-year LRC, PFS, and OS rates of 89, 82, and 89%, respectively, whereas the corresponding rates for the cetuximab-containing regimen were 85, 53, and 71% [285]. Beyond nab-PTX, other nanoparticles and nanosystems have also entered clinical evaluation in HNSCC. For example, the intratumoral injection of NBTXR3, a functionalized hafnium oxide nanoparticle-based radioenhancer, followed by intensity-modulated radiation therapy was feasible, with a favorable safety profile in patients unable to receive CDDP or cetuximab [290,291]. This approach showed promising results with an ORR of 80%, a complete response rate of 52%, a median PFS of 11.4 months, and a median OS of 18.1 months [290,291]. Moreover, the most common NBTXR3-related adverse events were stomatitis and tumor pain, with each occurring in 5% of the patients enrolled in the trial [291]. Anti-EGFR immunoliposomes loaded with doxorubicin were assessed for the treatment of advanced solid tumors, including HNSCC, leading to three cases of stable disease, one complete response, and one case of progressive disease, with one patient experiencing a grade 4 adverse event of anaemia [292]. NC-6004, a nanoparticle formulation of CDDP, was investigated in combination with gemcitabine in patients with advanced solid tumors [293]. Among the two HNSCC patients enrolled in the study, one achieved a partial response [293]. Furthermore, the combination therapy demonstrated an acceptable safety profile [293]. A liposomal formulation of CDDP combined with 5-FU was associated with lower rates of hematological toxicity than the corresponding regimen containing free CDDP, with the exception of grade 3 anemia [294]. In addition, grade 3 and 4 non-hematological adverse events occurred less frequently in patients receiving liposomal CDDP [294]. However, grade II renal toxicity was reported in 40% of patients treated with the liposomal formulation [294]. Furthermore, liposomal CDDP demonstrated lower efficacy, with a reduced objective partial remission rate (19 vs. 38.8%) and a higher proportion of patients experiencing disease progression (24 vs. 14.3%) compared with free CDDP [294]. Nevertheless, a higher percentage of patients achieved stable disease in the liposomal CDDP group than in the conventional CDDP group (64 vs. 50%) [294]. A different study evaluated liposomal CDDP in combination with radiotherapy in locally advanced HNSCC and found that the regimen was safe and the maximum tolerated dose could not be achieved [295]. Additionally, the treatment achieved an estimated OS rate of 41% and a disease-free survival rate of 25% [295]. Among the 20 patients enrolled, 11 achieved an initial complete response; however, 3 of these patients subsequently died from disease progression [295]. Furthermore, CDDP chitosan nanoparticles were first assessed in vivo, showing that they were more effective in reducing tumor volume compared with intravenous or intratumorally delivered CDDP [296]. Similarly, the topical transmucosal patch containing CDDP chitosan nanoparticles PRV111 also reduced tumor growth more effectively than the intraperitoneal delivery of CDDP [296]. In patients with oral cavity squamous cell carcinoma, PRV111 increased intratumoral CDDP concentrations and achieved a mean tumor volume reduction of 69% within approximately 7 days [296]. Clinical responses were observed in seven of eight participants (87.5%), while five participants (62.5%) experienced tumor shrinkage exceeding 90% [296]. These encouraging results further support the continuous investigation of nanocarrier systems for the treatment of HNSCC and OSCC, in particular. Nevertheless, the current evidence remains limited by the relatively small number of clinical trials and the modest sample sizes of many studies [286,297]. Furthermore, nab-PTX presents several advantages, including improved distribution, clearance properties, and the possibility of administration without steroid or antihistamine premedication. However, concerns regarding its safety profile remain, as conventional PTX is associated with potentially life-threatening hypersensitivity reactions [286,297]. For instance, in patients with pancreatic cancer, nab-PTX has been associated with increased hematological and hepatic adverse events [286,297]. Furthermore, a study involving patients with pancreatic and breast cancer suggested that nab-PTX may not reduce the risk of neutropenia, anemia, and hepatotoxicity compared with conventional PTX, contrary to previous assumptions [298]. However, these findings should be interpreted with caution, given the limited sample size of the study [298]. Collectively, these advances illustrate how physiologically relevant experimental models, multi-omics technologies, and nanotechnology-based platforms complement one another throughout the translational research pipeline in OSCC (Figure 3).

## 7. Conclusions and Future Perspectives

The landscape of OSCC research is undergoing a fundamental transformation driven by the integration of increasingly physiologically relevant experimental platforms with advanced molecular and computational tools [187,299]. No single model can fully recapitulate the biological, metabolic, and microenvironmental complexity of human OSCC. Instead, the complementary use of 2D and 3D in vitro systems, PDOs, microfluidic OoC platforms, ex vivo explants, and in vivo models, each capturing distinct aspects of tumor biology, offers a more comprehensive framework for mechanistic investigation and therapeutic development. A central theme emerging across these platforms is the critical role of metabolic reprogramming in OSCC progression, therapy resistance, and immune evasion. The Warburg effect, mitochondrial plasticity, and immunometabolic crosstalk consistently appear as key determinants of treatment failure, yet their accurate reproduction in experimental systems remains challenging. Advanced platforms, particularly organoids, OoC systems, and orthotopic in vivo models have shown a superior capacity to recapitulate metabolic gradients, hypoxic signaling, and microenvironment-driven resistance compared with conventional 2D systems. In parallel, nanotechnology-based delivery systems have emerged as powerful tools for targeted metabolic intervention in OSCC, enabling tumor-specific drug delivery, improved pharmacokinetics, and reduced systemic toxicity. The convergence of nanocarrier design with patient-derived experimental platforms offers a particularly promising avenue for the preclinical validation of precision therapeutic strategies. Given the diversity of currently available experimental platforms, selecting the most appropriate model depends on the specific biological question being addressed rather than on the assumption that a single system can reproduce all aspects of OSCC biology. Each model offers distinct strengths and limitations with respect to physiological relevance, experimental complexity, and translational applicability. Table 3 summarizes the experimental platforms that are best suited for different research objectives in OSCC research.

Looking ahead, the next frontier in OSCC research involves hybrid and complementary experimental strategies that combine the structural fidelity of organoids, the dynamic microenvironmental control of microfluidic systems, the systemic complexity of in vivo models, and the translational authenticity of comparative oncology platforms [300,301]. Advances in 3D bioprinting and tissue engineering further enhance this landscape by enabling the construction of multicellular tumor constructs that incorporate stromal elements, ECM components, and vascular-like structures [300,301]. The incorporation of artificial intelligence (AI), particularly for bioink optimization, real-time monitoring, and multi-omics-driven drug response prediction is accelerating the development of these platforms toward clinically actionable precision medicine tools [300,301]. This synergistic approach seeks to provide a high-resolution framework for identifying metabolic vulnerabilities and validating personalized therapies in a standardized and reproducible manner [173,190,301].

## Figures and Tables

**Table 1:** Comparative overview of in vitro experimental models used in OSCC research.

Model	Core Principle	Main Advantages	Best Suited to Study in OSCC	Limitations
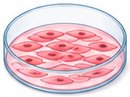 2D monolayer cultures	Cells grown in flat adherent monolayers	Low cost, high reproducibility, easy genetic manipulation, high-throughput compatible	Oncogenic signaling (*TP53*, PI3K/AKT/mTOR), basic metabolic pathways (Warburg effect), initial drug screening	Lack of 3D architecture, no ECM, no gradients, poor microenvironment representation, low translational relevance
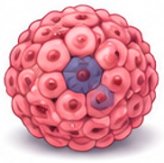 3D spheroids/scaffold-based cultures	Self-assembled multicellular aggregates or matrix-supported 3D systems	Recapitulate tumor architecture, generate physiological gradients, improved biological relevance	Hypoxia signaling (HIF activation), metabolic heterogeneity, EMT, invasion, drug penetration and resistance	Absence of vasculature, immune and full stromal components, variability in size and protocols
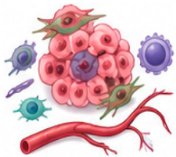 3D co-culture systems	Tumor cells combined with fibroblasts, immune, and endothelial cells	Model TME interactions and multicellularity	CAF-driven invasion, immune evasion, angiogenesis, tumor–stromal signaling, metabolic crosstalk	Increased complexity, lack of standardization, still incomplete representation of in vivo architecture
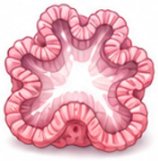 PDOs	3D structures derived from enzymatically dissociated patient tumor cells	Preserve patient-specific genetics, enable functional precision oncology, expandable ex vivo	Drug response prediction, inter- and intra-tumoral heterogeneity, metabolic plasticity, clonal evolution under therapy	Limited stromal/immune/vascular components (unless co-cultured), cost, technical variability
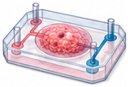 Microfluidic/OoC systems	Engineered microdevices with controlled flow and microenvironmental regulation	Mimic perfusion, shear stress, and dynamic gradients; real-time monitoring possible	Tumor–stroma and tumor–endothelium interactions, hypoxia-driven signaling, drug pharmacokinetics, therapy resistance under flow	Low throughput, technical complexity, limited standardization and scalability
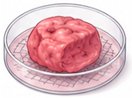 Ex vivo tissue explants	Fresh tumor tissue maintained intact in short-term culture	Preserve native architecture, ECM, immune and stromal components (short-term)	Tumor invasion, EMT, spatial heterogeneity, short-term drug and radiotherapy response	Short viability, progressive loss of function, high inter-sample variability

2D, two-dimensional; 3D, three-dimensional; CAF, cancer-associated fibroblasts; ECM, extracellular matrix; EMT, epithelial–mesenchymal transition; HIF, hypoxia-inducible factor; PDOs, patient-derived organoids; TME, tumor microenvironment; TP53, tumor protein p53 (*TP53* gene).

**Table 2:** Comparative overview of the principal in vivo experimental models used in OSCC research, highlighting their core biological characteristics, major advantages, most appropriate applications, and key limitations.

Model	Core Principle	Main Advantages	Best Suited to Study in OSCC	Limitations
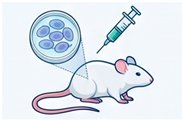 CDX	Implantation of human cancer cell lines into immunodeficient mice	High reproducibility, technical simplicity, and low cost	Early-stage drug evaluation and basic mechanistic studies of tumor biology	Lack of functional adaptative immune responses, species-specific stromal differences, potential subclone selection during in vitro expansion
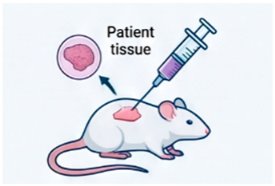 PDX	Direct implantation of patient tumor fragments into immunodeficient mice	Preserves histological architecture, intratumoral heterogeneity, and patient molecular diversity	Precision medicine application of therapeutic response and acquired resistance mechanisms	Variable engraftment rates depending on tumor pathology, absence of a functional human immune system
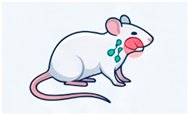 Orthotopic models	Implantation into the native anatomical site (tongue or oral mucosa)	Faithfully recapitulates tumor–stroma interactions, vascularization, and metastatic behavior	Investigation of local invasion, cervical lymph node involvement, and microenvironment-driven responses	High technical complexity requiring microsurgery, longitudinal monitoring is more difficult than in subcutaneous models
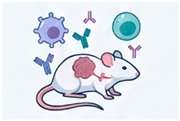 Humanized mouse models	Reconstitution of immunodeficient mice with human immune components (PBMC or HSC)	Enables investigation of tumor–immune interactions and immune checkpoint dynamics	Preclinical evaluation of immunotherapies (PD-1/PD-L1 inhibitors) and immunometabolic crosstalk	Risk of graft-versus-host disease (PBMC), extended engraftment periods (HSC), incomplete representation of human immune complexity
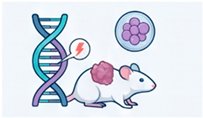 GEMMs	In situ tumor initiation via tissue-specific genetic alterations in immunocompetent hosts	Captures stepwise progression (dysplasia to carcinoma) in an intact immune and microenvironment	Early tumorigenesis, spontaneous disease evolution, and longitudinal tracking of metabolic adaptations	Histological differences between murine and human mucosa, incomplete capture of human interpatient mutational complexity, high costs and long development times
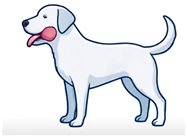 Comparative oncology models (COSCC)	Study of spontaneous OSCC in companion dogs	Naturally occurring tumors in immunocompetent hosts sharing environmental exposures with humans	Investigation of systemic metabolism, chronic inflammation, and conserved therapeutic vulnerabilities	Strict ethical standards and owner consent requirements, limited availability of standardized samples
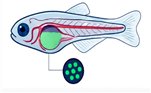 Zebrafish xenograft models	Microinjection of OSCC cells into transparent larvae or adult strains	Optical transparency allows real-time imaging of metabolic fluxes and early metastatic events	Rapid tumor phenotyping and real-time monitoring of angiogenesis, invasion, and metastatic dissemination, and metabolic dynamics	Significant tumor area variability, delivery challenges for hydrophobic compounds, lack of adaptive immunity in early larval stages
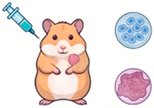 HBP model	Chemical induction in Syrian golden HBP.	Replicates the complete disease sequence with human-like histological and molecular characteristics	Experimental chemoprevention, radiotoxicity (mucositis), and longitudinal tissue recovery	Therapeutic response is size-dependent (poor remission for large tumors), buccal pouch lacks a direct anatomical human counterpart

CDX, cell line-derived xenograft; COSCC, canine oral squamous cell carcinoma; GEMM, genetically engineered mouse model; HBP, hamster buccal pouch; HSC, hematopoietic stem cells; OSCC, oral squamous cell carcinoma; PBMC, peripheral blood mononuclear cells; PD-1, programmed cell death protein 1; PD-L1, programmed death-ligand 1; PDX, patient-derived xenograft.

**Table 3:** Selection of experimental platforms according to the primary research objectives in OSCC.

Research Objective	Most Appropriate Platforms	Key Rationale
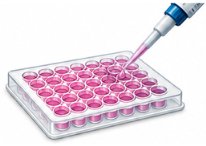 High-throughput drug screening	2D Monolayer	2D offers low cost and high-throughput compatibility
	Zebrafish	Zebrafish allow for rapid, cost-efficient in vivo phenotypic screening and early dissemination tracking
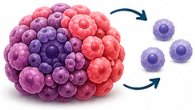 Mechanisms of therapy resistance	PDOs/3D Spheroids/PDX	3D models and PDOs recreate the oxygen/nutrient gradients and metabolic heterogeneity that drive resistance. PDXs preserve the acquired resistance mechanisms seen in patients
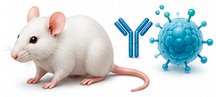 Immunotherapy evaluation	Humanized mice/GEMMs	GEMMs provide a fully intact immunocompetent microenvironment. Humanized mice allow for the study of human-specific immune checkpoint (PD-1/PD-L1) dynamics
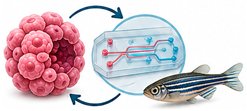 Tumor metabolism and metabolic rewiring	Organoids/OoC/Zebrafish/GEMMs	OoC allows for precise control of spatiotemporal metabolic gradients. Zebrafish provide unique real-time imaging of metabolic fluxes. GEMMs track metabolic shifts from initiation to metastasis
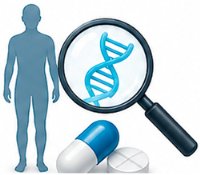 Precision medicine	PDOs/PDX	PDOs and PDXs preserve patient-specific genetics and histopathology, allowing for individualized drug response prediction and biomarker validation

2D, two-dimensional; 3D, three-dimensional; GEMM, genetically engineered mouse model; OoC, organ-on-chip; PD-1, programmed cell death protein 1; PD-L1, programmed death-ligand 1; PDOs, patient-derived organoids; PDX, patient-derived xenograft.

## Data Availability

No new data were created or analyzed in this study. Data sharing is not applicable to this article.

## Abbreviations

The following abbreviations are used in this manuscript:

2D	Two-dimensional
2-DG	2-deoxyglucose
2-PG	2-phosphoglycerate
3D	Three-dimensional
3-PG	3-phosphoglycerate
4-NQO	4-nitroquinoline-1-oxide
5-FU	5-fluorouracil
AI	Artificial intelligence
AT	Ataxia-telangiectasia
Bmi-1	B cell-specific Moloney murine leukemia virus integration site 1
BRAF	B-Raf proto-oncogene
CAF	Cancer-associated fibroblast
CBX3	Chromobox protein homolog 3
CDCP1	CUB domain-containing protein 1
CDDP	Cisplatin
CDKN2A	Cyclin-dependent kinase inhibitor 2A
CDT	Chemodynamic therapy
CDX	Cell line-derived xenografts
COSCC	Canine oral squamous cell carcinoma
CRISPR	Clustered regularly interspaced short palindromic repeats
CTLA	Cytotoxic T-lymphocyte-associated protein
CXC	C-X-C motif chemokine
DCA	Dichloroacetate
DGKG	Diacylglycerol kinase gamma
DNA	Deoxyribonucleic acid
DPPA	Dipalmitoyl phosphatidic acid
ECM	Extracellular matrix
EGFR	Epidermal growth factor receptor
EMT	Epithelial–mesenchymal transition
GEMM	Genetically engineered mouse model
GLUT	Glucose transporter
GPX4	Glutathione peroxidase 4
GSTO2	Glutathione *S*-transferase omega 2
HBP	Hamster buccal pouch
HIF-1a	Hypoxia-inducible factor-1 alpha
HNSCC	Head and neck squamous cell carcinoma
HPV	Human papillomavirus
HSC	Hematopoietic stem cells
IC_50_	Inhibition concentration 50
IGF1	Insulin-like growth factor 1
IL1RN	Interleukin 1 receptor antagonist
ITG	Integrin subunit
KMT2D	Lysine methyltransferase 2D
KRT	Keratin
KRAS	Kirsten rat sarcoma viral proto-oncogene
MCT	Monocarboxylate transporters
MEF2A	Myocyte enhancer factor 2A
MMP	Matrix metalloproteinase
NIR	Near-infrared radiation
NLC	Nanostructured lipid carriers
NRF2	Nuclear factor erythroid 2-related factor 2
OoC	Organ-on-chip
ORR	Objective response rate
OS	Overall survival
OSCC	Oral squamous cell carcinoma
PBMC	Peripheral blood mononuclear cells
PD-1	Programmed cell death protein 1
PD-L1	Programmed death-ligand 1
PDO	Patient-derived organoid
PDT	Photodynamic therapy
PDX	Patient-derived xenograft
PEG	Polyethylene glycol
PEI	Polyethylenimine
PEP	Phosphoenolpyruvate
PIK3CA	Phosphatidylinositol-4,5-bisphosphate 3-kinase catalytic subunit alpha
PLA	Poly(D,L-lactic acid)
PLGA	Poly(lactic-co-glycolic acid)
PMVEMA	Poly(methyl vinyl ether alt maleic anhydride)
PTX	Paclitaxel
ROS	Reactive oxygen species
SASH1	*S*-adenosyl-methionine and SH3 domain-containing protein 1
SLNs	Solid lipid nanoparticles
TCGA	The Cancer Genome Atlas
TME	Tumor microenvironment
TP53	Tumor protein p53
TRAIL	TNF-related apoptosis-inducing ligand
Trp53	Transformation related protein 53
ULA	Ultra-low attachement
USP30	Ubiquitin-specific protease 30
ZEB2	Zinc finger E-box binding homeobox 2
ZIF-8	Zeolitic imidazolate framework-8
